# An Epigenetically Remodeled EMT–Inflammatory Clone Associated with Spatial Immune Exclusion in Clear Cell Renal Cell Carcinoma

**DOI:** 10.3390/ijms27219668

**Published:** 2026-10-28

**Authors:** Rifang Lan, Lixia Weng, Bingbing Cao, Aotuo Li, Jingyi Zhang, Hongbo Shi, Lei Yang, Yingli Lv

**Affiliations:** College of Bioinformatics Science and Technology, Harbin Medical University, Harbin 150081, China; 2023020632@hrbmu.edu.cn (R.L.); 2024020600@hrbmu.edu.cn (L.W.); 2024020594@hrbmu.edu.cn (B.C.); 2025020444@hrbmu.edu.cn (A.L.); 2025020525@hrbmu.edu.cn (J.Z.); shihongbo@ems.hrbmu.edu.cn (H.S.)

**Keywords:** clear cell renal cell carcinoma, single-cell multi-omics, malignant cell subpopulation, epigenetic regulation, spatial immune exclusion

## Abstract

Clear cell renal cell carcinoma (ccRCC) exhibits intra-tumoral heterogeneity and spatial complexity that restrict immune infiltration, yet the malignant populations driving spatial immune exclusion remain elusive. We integrated scRNA-seq, paired scATAC-seq, spatial transcriptomics, and multiple bulk cohorts to identify the malignant subpopulation driving ccRCC progression. Multi-omics analyses, including malignancy inference, epigenomic profiling, cell–cell communication, and spatial modeling, characterized its molecular features and clinical relevance. We identified a malignant subpopulation co-activating epithelial–mesenchymal transition (EMT) and inflammatory programs, termed the aggressive EMT–inflammatory clone (AEIC). AEIC exhibited pronounced genomic instability, enhanced stemness, and poor prognosis across multicenter cohorts (*p* < 0.001). Chromatin accessibility revealed a *MAZ/RUNX1*-centered network driving key AEIC signature genes such as *TGFBI* and *COL6A2*. Cell–cell communication analysis suggested AEIC interacts with NK_CD16− cells via the COL6A2–(ITGA1+ITGB1) axis. Spatial analyses showed AEIC-enriched regions forming an extracellular matrix-rich barrier associated with NK_CD16− cell exclusion, M2-like macrophage polarization, and increased genomic instability. Finally, an AEIC-derived 16-gene signature stratified prognosis across independent cohorts and was associated with distinct drug-sensitivity patterns, including increased predicted sensitivity to Bortezomib and AZD4547. Overall, AEIC is associated with spatial and epigenetic remodeling in ccRCC, and the AEIC-derived signature supports prognostic stratification and further exploration of potential therapeutic vulnerabilities associated with the AEIC phenotype.

## 1. Introduction

Clear cell renal cell carcinoma (ccRCC), the most common histological subtype of kidney cancer, accounts for 70–80% of all renal malignancies and remains a major cause of cancer-related mortality worldwide [1,2,3,4]. Although immune checkpoint inhibitors and targeted therapies have substantially improved the management of advanced ccRCC, durable [1,4] clinical benefit remains limited for a considerable proportion of patients. Approximately 30% of patients exhibit primary resistance or experience postoperative recurrence, highlighting an incomplete understanding of the biological mechanisms underlying disease progression and therapeutic failure [5,6,7].

Intra-tumoral heterogeneity (ITH), a defining hallmark of ccRCC, contributes substantially to therapeutic resistance and poor clinical outcomes [1,4]. However, conventional bulk RNA-seq averages transcriptional signals across heterogeneous cell populations, limiting its ability to identify the malignant cell subpopulations responsible for tumor progression and treatment resistance [8,9,10,11,12]. Consistent with this limitation, some patients predicted to have a favorable prognosis based on bulk transcriptome profiles still develop early metastatic disease, suggesting the presence of rare but highly aggressive malignant cell populations within the primary tumor. Recent advances in single-cell and spatial transcriptomics have revealed that distinct malignant cell subpopulations are distributed unevenly across the tumor and dynamically interact with the surrounding tumor microenvironment [13,14,15]. This spatial heterogeneity is reflected histologically by a non-uniform distribution of immune cells across the tumor, with some regions, such as tertiary lymphoid structures, showing dense immune infiltration, whereas others, particularly at the tumor–normal interface, remain immune-excluded or locally immunosuppressive [13,14,15]. Emerging evidence suggests that specific malignant cell subpopulations cooperate with cancer-associated fibroblasts, tumor-associated macrophages, and regulatory T cells to establish localized immunosuppressive niches associated with immune exclusion, therapeutic resistance, and poor clinical outcomes [16,17,18].

The malignant cell subpopulation responsible for establishing this immune-excluded niche remains unknown [16,17,19,20,21]. Moreover, the epigenetic mechanisms that maintain its aggressive phenotype and enable remodeling of the surrounding stroma remain poorly understood [17]. Single-cell assay for transposase-accessible chromatin using sequencing (scATAC-seq) enables genome-wide profiling of chromatin accessibility and identifies regulatory programs governing cell-state transitions [22,23,24,25]. In parallel, spatial transcriptomics preserves tissue architecture while mapping gene expression to its spatial context, enabling in situ characterization of cell–cell interactions [25,26,27]. However, studies integrating these approaches to link the epigenetic regulation of malignant cell subpopulations with spatial immune exclusion in ccRCC remain scarce [20,21].

In this study, we integrated single-cell RNA sequencing (scRNA-seq), scATAC-seq, spatial transcriptomics, and bulk transcriptome datasets to identify the malignant cell subpopulation responsible for spatial immune exclusion in ccRCC. We systematically characterized its molecular, epigenetic, and spatial features, uncovered the regulatory programs underlying its aggressive phenotype, and assessed its clinical significance across independent patient cohorts. These findings provide mechanistic insights into how epigenetic regulation contributes to the malignant cell subpopulation underlying spatial immune exclusion and provide a framework for risk stratification and therapeutic exploration in ccRCC.

## 2. Results

### 2.1. Single-Cell Transcriptomic Atlas of ccRCC Resolves the Multicellular Ecosystem and Microenvironmental Heterogeneity

**Figure 1:**
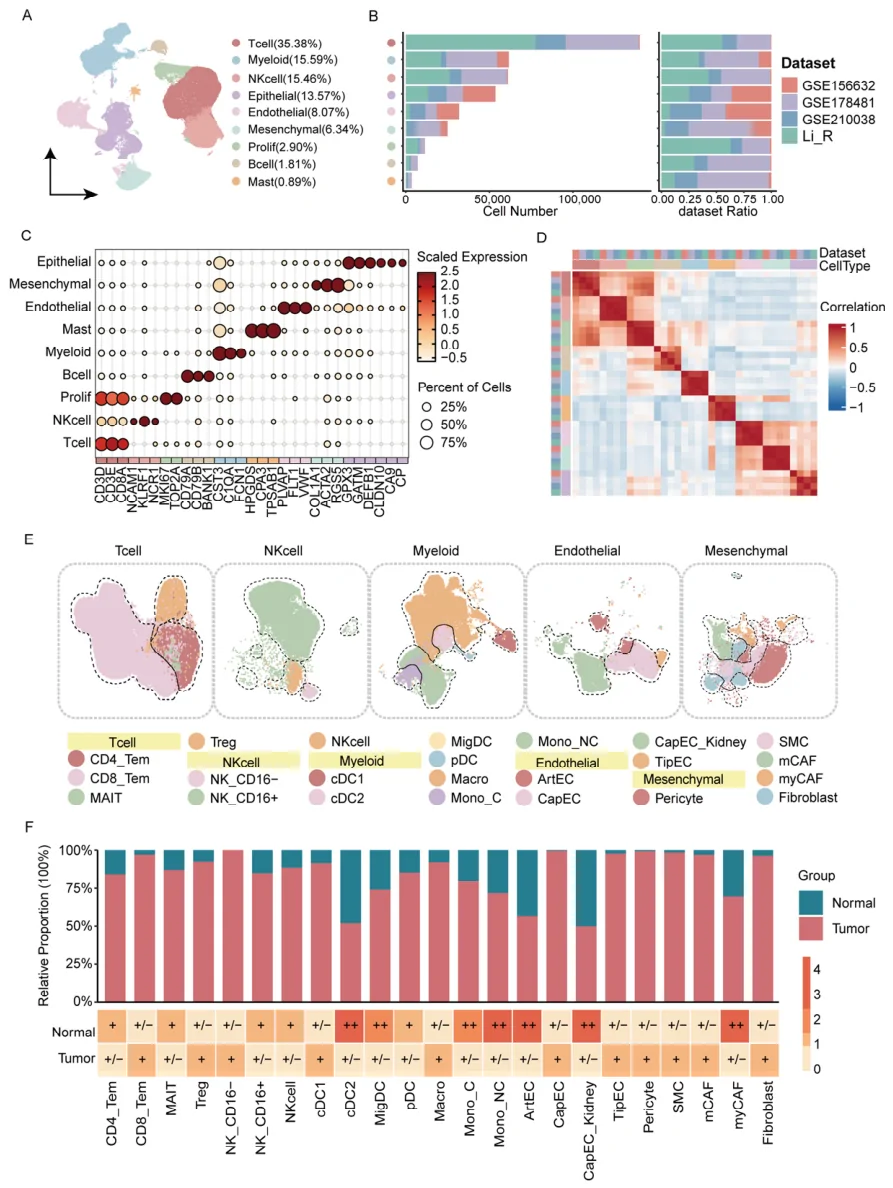
Single-cell transcriptomic atlas of ccRCC resolves the multicellular ecosystem and microenvironmental heterogeneity. (**A**) Uniform manifold approximation and projection (UMAP) plot showing the nine major cell lineages identified based on canonical marker genes. (**B**) Bar plots showing the absolute cell counts (left) and proportions (right) of each cell lineage across datasets. (**C**) Dot plot showing the expression patterns of canonical marker genes for each cell lineage; dot size represents the percentage of expressing cells, and color intensity represents the average expression level. (**D**) Heatmap of Pearson correlation coefficients showing the expression consistency of identical cell lineages across datasets. (**E**) UMAP plot showing the 23 refined non-epithelial subpopulations. (**F**) Bar plot (top) and Ro/e enrichment heatmap (bottom) showing the distribution preferences of non-epithelial subpopulations across tumor and normal tissues. ++ and + represent significant enrichment (Ro/e > 1.5 and > 1, respectively); +/− represents no significant enrichment (Ro/e ≤ 1).

Our study incorporated scRNA-seq data from four independent cohorts (Table S1). After quality control and harmony [28] batch correction, we constructed the atlas of ccRCC comprising 396,131 high-quality cells (Figure S1A,B, Section 4). Quantitative integration benchmarks confirmed the effective removal of technical batch effects while preserving biological signals, with low dataset-label driven clustering (ARI = 0.092, NMI = 0.101, dataset-label Silhouette width = −0.015) and high inter-dataset mixing (batch mixing score = 0.667). Non-linear dimension reduction and unsupervised clustering identified nine major cell lineages based on canonical marker gene expression. T cells (35.38%), myeloid cells (15.59%), NK cells (15.46%), and epithelial cells (13.57%) constituted the largest cell populations (Figure 1A–C and Figure S1C). Cross-dataset Pearson correlation analysis demonstrated high concordance of lineage annotations across cohorts (Figure 1D).

To further characterize the non-epithelial compartment, we performed subclustering analysis combined with differential expression analysis and CellTypist annotation [29], identifying 23 distinct subpopulations (Figure 1E and Figure S2A,C–F). SCENIC analysis [30] further supported the T-cell annotations at the gene regulatory level (Figure S2B). Tissue distribution analysis showed that Tregs, CD8_Tem, NK_CD16− cells, myofibroblastic cancer-associated fibroblast (mCAF), capillary endothelial cell (CapEC) and tip endothelial cell (TipEC) were coordinately enriched in tumor tissues (Ro/e > 1), indicating robust cross-lineage synergy within the microenvironment (Figure 1F).

### 2.2. Identification of AEIC as a Core Malignant Cell Subpopulation with Adverse Clinical Impact

**Figure 2:**
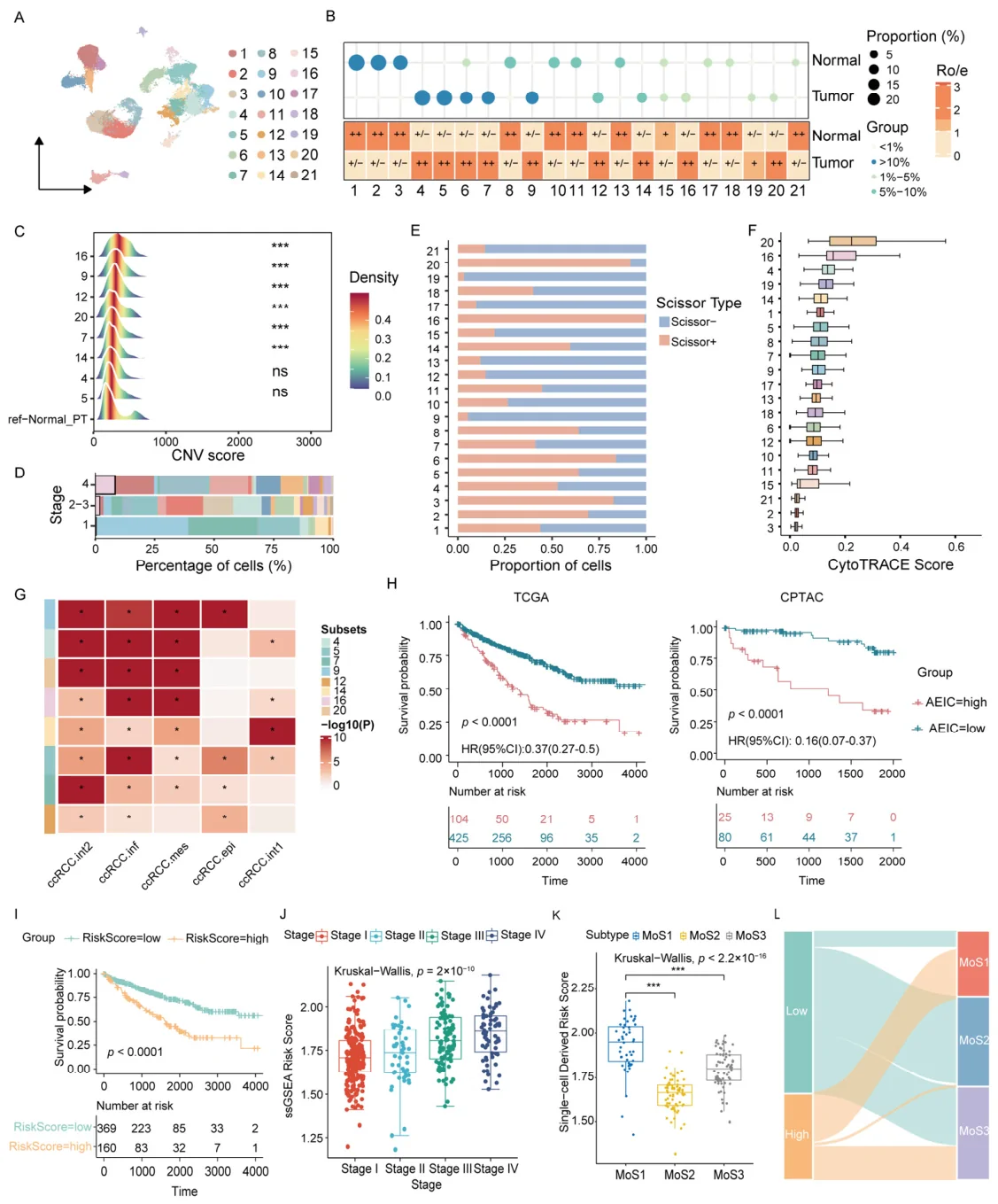
Identification of AEIC as a core malignant cell subpopulation with adverse clinical impact. (**A**) UMAP plot showing the re-clustering of epithelial subclusters. (**B**) Proportions (top) and Ro/e enrichment (bottom) of each subcluster across tissue tumor and normal tissues. ++ and + represent significant enrichment (Ro/e > 1.5 and > 1, respectively); +/− represents no significant enrichment (Ro/e ≤ 1). (**C**) InferCNV-estimated genomic instability scores of each subcluster (***, *p* < 0.001; ns, not significant). (**D**) Proportion shifts of key malignant subclusters across clinical stages. (**E**) Distribution of prognosis-associated (Scissor+) cells across epithelial subclusters. (**F**) CytoTRACE differentiation potential scores of each subcluster (*p* < 0.001). (**G**) Activity differences of epithelial subclusters across canonical metabolic pathways(*, *p* < 0.05). (**H**) OS analysis of AEIC abundance in the TCGA and CPTAC cohorts. (**I**) Kaplan–Meier overall survival curves for TCGA-KIRC patients stratified by the 43-gene AEIC-signature risk scores. (**J**) Box plot showing the distribution differences of the AEIC-signature risk scores across distinct pathological stages. (**K**) Box plot showing the distribution of the AEIC risk scores across three MoS subtypes (Kruskal–Wallis test, ***, *p* < 0.001). (**L**) Sankey diagram illustrating the mapping relationship between AEIC risk groups (High/Low) and consensus MoS molecular subtypes (MoS1-3).

To investigate the malignant heterogeneity within the epithelial compartment, we re-clustered the epithelial lineage and identified 21 distinct subclusters (Figure 2A and Figure S3A, Table S2). Ro/e analysis identified clusters 9, 12, 16, and 20 as tumor-enriched epithelial subpopulations (Ro/e > 2, Table S3). These clusters specifically overexpressed canonical ccRCC markers and oncogenes, including *CA9*, *TGFA*, and *ENPP3* (Figure 2B and Figure S3B). Cluster 16 exhibited the highest copy number variation (CNV) score inferred by inferCNV [31], significantly higher than that of the other tumor-enriched subclusters (*p* < 0.001, Figure 2C, Section 4). Among all malignant epithelial subclusters, only cluster 16 showed a progressive increase in abundance with advancing pathological stage (Figure 2D). Together, these findings identified cluster 16 as the candidate malignant cell subpopulation for subsequent analyses.

We next characterized the clinical and biological features of this candidate subpopulation. Integration of follow-up survival data from the TCGA-KIRC cohort using the Scissor algorithm [32] showed that cluster 16 harbored the highest proportion of Scissor+ cell states associated with unfavorable prognosis (Figure 2E). CytoTRACE analysis [33] further revealed that its stemness score was second only to cluster 20 (Figure 2F). Metabolic pathway analysis showed increased energetic metabolism in cluster 16 (Figure S3C). External consensus molecular subtype mapping demonstrated that cluster 16 uniquely recapitulated the molecular features of both the mesenchymal (ccRCC.mes) and inflammatory (ccRCC.inf) subtypes (*p* < 0.05, Figure 2G, Section 4). Functional enrichment analysis further showed prominent activation of hallmark pathways, including EMT, coagulation cascades, and complement responses (Figure S3D). Based on these molecular and biological features, we designated cluster 16 as the AEIC.

We next established an AEIC signature by combining expression specificity scoring with iterative optimization, yielding a 43-gene signature (Figure S3E,F, Table S4). To evaluate the clinical relevance of AEIC at the population level, we applied BayesPrism deconvolution across the TCGA-KIRC and CPTAC cohorts (Figure S4A), which demonstrated that high AEIC abundance was associated with significantly poorer OS (*p* < 0.001, Figure 2H). High AEIC abundance was also associated with increased Treg infiltration, higher chromosomal instability scores, and elevated tumor mutation burden (Figure S4B,C).

To determine whether the malignant characteristics of cluster 16 are distinct from those of other epithelial subclusters, we performed systematic comparisons across multiple dimensions. First, module scoring based on the AEIC signature demonstrated that cluster 16 exhibited significantly higher expression scores than all other subclusters (*p* < 2 × 10^−16^, Figure S4D), confirming the preferential enrichment of this signature in cluster 16. Second, GO enrichment analysis revealed that cluster 16 was predominantly enriched in hypoxia response and wound healing, whereas cluster 4 was biased toward coagulation, cluster 12 favored energy metabolism, and cluster 14 was enriched in PDGF signaling (Figure S4E), indicating that distinct subclusters execute distinct biological programs. Finally, at the clinical level, univariate Cox regression and Spearman correlation analyses established that cluster 16 abundance uniquely conferred adverse prognosis across both cohorts (TCGA HR = 1.45; CPTAC HR = 1.83, both *p* < 0.001) and positively correlated with pathological stage, grade, and OS events, whereas cluster 14 and cluster 12 displayed protective or inverse associations (Figure S4F–I). Together, these findings confirm that the adverse prognostic impact of cluster 16 stems from its specific molecular and functional attributes rather than generalized properties shared across the epithelial lineage.

Finally, we further characterized the clinical and molecular subtype associations of the AEIC signature in the TCGA-KIRC cohort. Single-sample gene set enrichment analysis (ssGSEA) revealed that high AEIC signature scores in the TCGA-KIRC cohort were significantly associated with poor prognosis and advanced pathological stage (*p* < 0.001, Figure 2I,J). Patients with high AEIC scores predominantly belonged to the highly aggressive MoS1 subtype (Figure 2K,L), supporting the clinical applicability of the AEIC signature.

### 2.3. Epigenomic Reprogramming by MAZ/RUNX1-Centered Regulators Sustains the Aggressive Phenotype of AEIC

**Figure 3:**
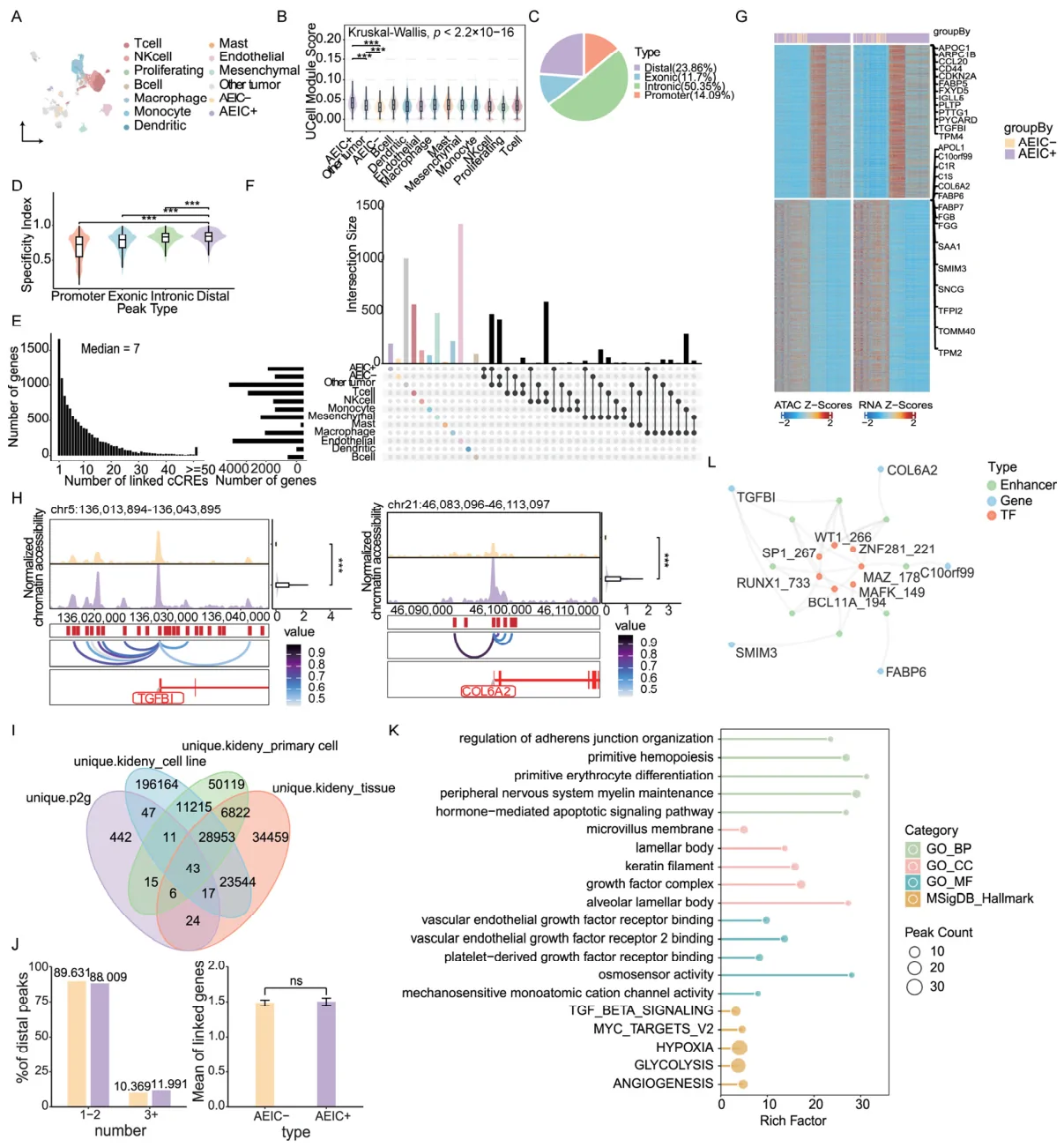
Cis-regulatory landscape and epigenomic reprogramming of the AEIC subpopulation. (**A**) UMAP plot showing the panoramic landscape of scATAC-seq data. (**B**) Distribution of AEIC feature scores across cell lineages (***, *p* < 0.001). (**C**) Pie chart showing the distribution proportions of significant cis-regulatory elements (CREs) across genomic functional zones. (**D**) Violin plot comparing the cell-specificity indices of regulatory elements across different functional zones (***, *p* < 0.001). (**E**) Number distribution of candidate CREs associated with each target gene. (**F**) UpSet plot showing the distribution of P2G-associated genes across cell lineages. (**G**) Heatmap of significant P2G associations between AEIC+ and AEIC- subpopulations. (**H**) Genomic browser tracks and violin plots showing the chromatin accessibility and expression levels of key marker genes *TGFBI* and *COL6A2* (***, *p* < 0.001). (**I**) Venn diagram showing the intersection between AEIC+ candidate elements and ENCODE normal kidney background data. (**J**) Bar plot comparing the number of target genes associated with AEIC+ and AEIC− specific distal elements (ns, not significant). (**K**) Functional enrichment analysis of specific dREs (*p* < 0.001). (**L**) The “transcription factor–enhancer–target gene” regulatory network of the AEIC+ subpopulation.

To investigate the epigenetic basis underlying the aggressive phenotype of AEIC, we integrated matched scRNA-seq and scATAC-seq datasets through cross-modal label transfer, generating a high-resolution atlas linking transcriptomic states with chromatin accessibility (Figure 3A, Section 4). The inferred AEIC+ cell population retained significantly higher AEIC signature scores than other cell populations at the chromatin accessibility level (*p* < 2.2 × 10^−16^, Figure 3B), demonstrating concordance between transcriptional identity and chromatin accessibility. Genome-wide Peak-to-Gene (P2G) analysis identified 918,246 significant regulatory links (FDR < 0.01), with more than 70% located in non-coding regions (Figure 3C). Among the 111,213 high-confidence links (correlation coefficient > 0.5), distal regulatory elements (dREs) exhibited the highest cell-type specificity, as indicated by the *Tau* index (*Tau* index = 0.832, *p* < 0.001, Figure 3D). On average, each target gene was associated with more than seven regulatory elements, indicating a predominant many-to-one regulatory architecture (Figure 3E). Consistently, the cis-regulatory network of AEIC+ cells showed limited overlap with those of other cell populations (Figure 3F), supporting the presence of an independent regulatory program.

To identify the specific distal elements driving this AEIC+ regulatory program, we next compared distal P2G links between AEIC+ and AEIC− cells. Transcriptional expression levels in AEIC+ cells showed high regulatory coordination with the accessibility of their associated peaks (Figure 3G). Distal peaks that were specifically accessible in AEIC+ cells and strongly correlated with target gene expression were defined as putative dREs. For core AEIC genes, including *TGFBI* and *COL6A2*, the corresponding dREs exhibited markedly higher chromatin accessibility in the AEIC+ cells, accompanied by elevated mRNA expression (Figure 3H). After excluding regulatory elements shared with normal kidney tissue using ENCODE reference data, 442 AEIC+-specific dREs were retained (Figure 3I, Table S5, Section 4). These dREs displayed a higher proportion of one-to-many regulatory relationships (Figure 3J) and were enriched in hypoxia-response and TGF-β signaling pathways (Figure 3K). Notably, 107 of these dREs (24.21%) significantly overlapped with tumor H3K27ac peaks (*p* < 0.001) in the independent clinical cohort GSE309697 (Figure S5A), indicating active enhancer marks in a subset of AEIC+-specific dREs.

To identify the upstream transcription factors (TFs) regulating AEIC+-specific dREs, we implemented a multi-step convergence screening strategy. Motif annotation identified 896 candidate TFs, whereas chromVAR analysis detected 216 motifs with significantly increased accessibility in AEIC+ cells compared with AEIC− cells (FDR < 0.05, MeanDiff > 0). By integrating these two independent screening outputs, we narrowed the candidate pool down to 80 high-confidence TFs, which were further prioritized using the MCRS framework (Materials and Methods). This analysis identified seven core TFs, with *MAZ* and *RUNX1* ranking highest (Figure S5B, Table S6). All seven TFs showed increased transcriptional activity in AEIC+ cells (Figure S5C), and genomic footprinting analysis further confirmed enhanced motif occupancy for most TFs (Figure S5D). In contrast, the transcriptional repressor *BCL11A* exhibited reduced chromatin accessibility in AEIC+ cells (Figure S5D). Finally, we constructed a regulatory network linking TFs, enhancers, and their target genes, which highlighted key regulatory axes including the *RUNX1*–enhancer–*TGFBI* axis (Figure 3L, Table S7).

We next validated the core TF–target gene regulatory axes (Table S7) using the independent bulk cohort GSE309697 (Materials and Methods). For all 442 dREs, 73 dREs (16.52%) exhibited significantly increased accessibility in tumor compared with normal (*p* < 0.05, log2FC > 0, Table S8), whereas 6 dREs (1.36%) showed decreased accessibility and 363 dREs (82.13%) showed no significant difference. Among the core TF–target gene axes, the *MAZ*-targeting *COL6A2* locus (*MAZ*_178|*COL6A2*) exhibited significantly higher openness in tumor compared with normal (*p* = 0.016, Figure S5E), consistent with the single-cell observation of elevated *MAZ* motif accessibility. The *BCL11A*-targeting *TGFBI* locus showed a decreasing trend in tumor (*p* = 0.082). In contrast, *RUNX1*-targeting *TGFBI* loci did not show significant changes (Figure S5E). At the H3K27ac level, 2 of the 8 core enhancer regions (25.0%) overlapped with tumor H3K27ac peaks, comparable to the global dREs (Fisher’s exact test *p* = 1.0, Figure S5A). Both overlapped regions regulate *TGFBI* and involve binding motifs for *MAZ*, *BCL11A*, and *RUNX1*. Moreover, public ChIP-seq analysis across three repositories (Cistrome DB, ENCODE, GEO) supported the potential direct binding of *MAZ*/*RUNX1* to these dREs (Table S9).

### 2.4. AEIC Establishes Intercellular Communication with NK_CD16− Cells via the COL6A2 Signaling Axis

**Figure 4:**
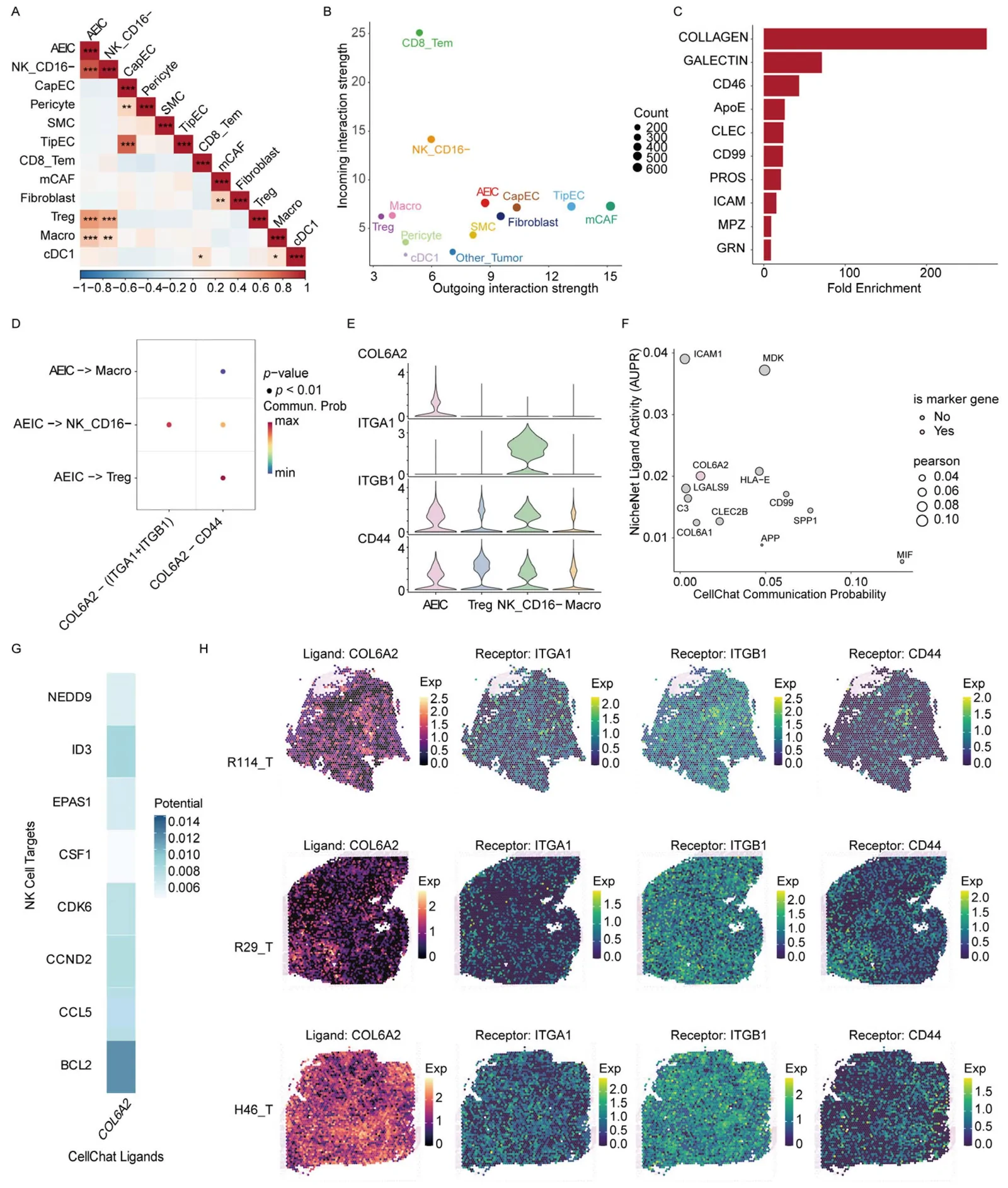
COL6A2-mediated communication between AEIC and NK_CD16− cells. (**A**) Co-occurrence network of tumor microenvironment components (***, *p* < 0.001; **, *p* < 0.01; *, *p* < 0.05). (**B**) CellChat analysis of cell communication intensity. (**C**) Comparison of communication pathway probabilities between AEIC and other tumor subclusters. (**D**) Communication intensities of ligand–receptor pairs between the AEIC subpopulation and -specific immune cells. (**E**) Expression distribution of core ligand–receptor genes across target subpopulations. (**F**) Evaluation of candidate ligands from AEIC to NK_CD16− cells. (**G**) Regulatory potential of COL6A2 on downstream target genes in NK_CD16− cells. (**H**) Spatial expression of *COL6A2*, *ITGA1*, *ITGB1*, and *CD44* across tissue sections.

To characterize the synergistic relationships between AEIC and immune cells within the tumor microenvironment, we first evaluated abundance correlations between AEIC and 11 tumor-enriched non-epithelial subpopulations (Materials and Methods). AEIC exhibited the strongest positive correlation with NK_CD16− cells (r = 0.8142), together with significant positive correlations with Tregs and macrophages (Figure 4A). CellChat analysis [34] further showed that AEIC exhibited stronger outgoing signaling activity than other malignant subpopulations. Specifically, AEIC acted as an active signal sender in autocrine feedback as well as in outgoing signaling to NK_CD16−, Treg, and macrophage populations (Figure 4B and Figure S6A). Pathway enrichment analysis identified COLLAGEN, GALECTIN, and CD46 as the major signaling pathways enriched in AEIC (Figure 4C). Within the COLLAGEN pathway, AEIC contributed substantially more outgoing signaling than other malignant subpopulations (Figure S6B,C), indicating enhanced collagen-mediated communication between AEIC and surrounding immune cells.

To further dissect the molecular basis of these communications, we interrogated the core genes of AEIC and identified two candidate ligand–receptor axes: COL6A2–(ITGA1+ITGB1) and COL6A2–CD44 (Figure 4D). *COL6A2* was highly expressed in AEIC, whereas *ITGA1* was predominantly expressed in NK_CD16− cells. In contrast, *ITGB1* and *CD44* were detected in both cell populations (Figure 4E). NicheNet analysis [35] identified COL6A2 as the only core ligand with both high communication strength and predicted regulatory activity. Its predicted downstream targets included genes involved in NK_CD16− cell survival and cell-cycle regulation, including *CCND2*, *BCL2*, and *ID3* (Figure 4F,G). Spatial transcriptomic analysis demonstrated consistent co-localization of COL6A2 with its receptors *CD44*, *ITGB1*, and *ITGA1* across tissue sections (Figure 4H and Figure S7). Together, these findings support the involvement of the COL6A2 signaling axis in AEIC–NK_CD16− cell communication.

### 2.5. Spatial Transcriptomics Confirms That AEIC Core Zones Construct a Physical Barrier Mediating NK_CD16− Cell Exclusion

**Figure 5:**
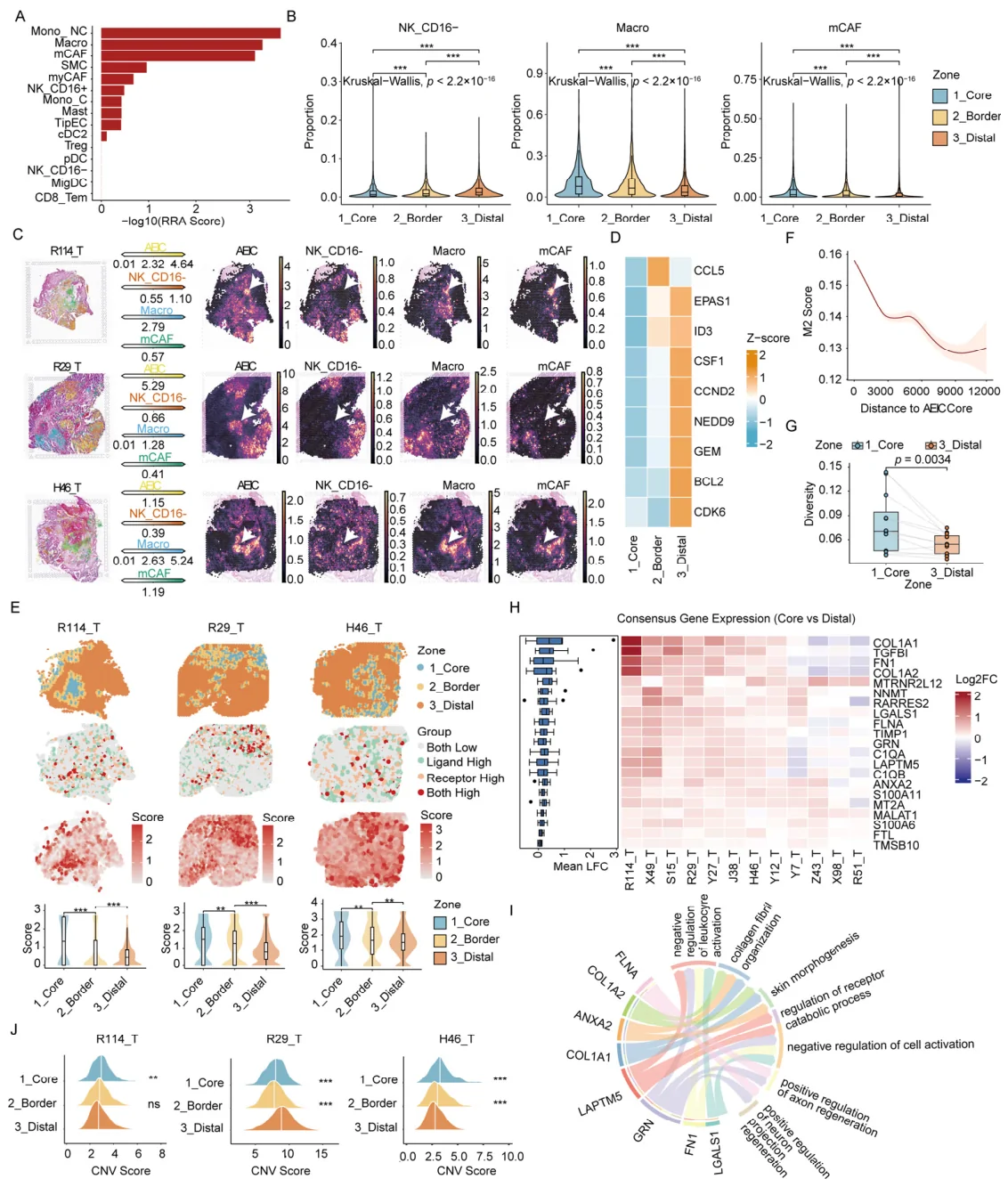
Spatial transcriptomic validation of AEIC core zone barrier and NK_CD16− cell exclusion. (**A**) Cross-sample rank integration of spatial distributions for cell subpopulations around the tumor core. (**B**) Quantitative abundance comparison of NK_CD16−, Macro, and mCAF populations across different spatial zones (***, *p* < 0.001). (**C**) In situ distribution maps of AEIC, Macro, mCAF, and NK_CD16− cells on tissue sections. (**D**) Spatial expression of NK_CD16− target genes within tissue sections. (**E**) Spatial anatomical zone definitions (top), distribution characteristics of COL6A2–CD44 interaction intensity (middle), and quantitative comparison of interaction scores across spatial zones (bottom) (Wilcoxon test, ***, *p* < 0.001; **, *p* < 0.01). (**F**) GAM fitting curves showing the spatial gradient of macrophage M2 polarization scores. (**G**) Comparison of transcriptomic diversity across spatial zones. (**H**) Heatmap of cross-sample consensus differentially expressed genes between the core and distal zones. (**I**) Functional enrichment analysis of consensus differentially expressed genes. (**J**) Ridge plot showing the quantitative comparison of CNV scores across spatial zones (***, *p* < 0.001; **, *p* < 0.01; ns, not significant).

To resolve the spatial distribution of AEIC and its impact on the microenvironment in situ, we partitioned tumor tissues into core, border, and distal zones based on AEIC abundance (Materials and Methods). Cross-sample integration using the robust rank aggregation (RRA) algorithm [36] revealed that non-classical monocytes (Mono_NC), macrophages, and mCAF were enriched in the AEIC core zone (−log10(RRA Score) = 3.79, 3.41, 3.25). In contrast, NK_CD16− cells displayed an exclusion trend, showing the lowest proportion in the core zone (−log10(RRA Score) = 0) and gradually increasing with distance from the core (Figure 5A,B). These results indicated that a local microenvironment dominated by myeloid cells and fibroblasts formed around the AEIC core zone, whereas NK_CD16− cells were relatively deficient in this region. Consistently, spatial analysis of 16 functional markers for immune effector cells revealed attenuated effector/activation (*IL15*, *IL7*, *CXCR3*) and inhibitory/exhaustion (*PDCD1*, *LAG3*, *HAVCR2*) gene expression in the core zone relative to distal regions across the 12 samples (Figure S8A). Module-level scoring further confirmed that both activation and exhaustion signatures exhibited higher spatial spot coverage (pos_pct) and elevated overall signal intensity (mean_score) in distal areas compared to the AEIC core (Figure S8B,C), confirming the spatial restriction of immune effector cell infiltration and functional activity.

To further validate the AEIC–NK_CD16− interaction predicted by scRNA-seq analysis, we examined in situ cellular distribution and molecular expression gradients. Spatial mapping revealed co-localization among AEIC, macrophages, and mCAF, whereas NK_CD16− cells exhibited a distinct spatial distribution (Figure 5C and Figure S10). Spatial patterns of NicheNet-predicted NK_CD16− target genes, including *CCL5* and *IDO1*, showed high expression in the distal zone with gradient attenuation near the core (Figure 5D). Analysis of the core ligand–receptor pairs COL6A2–CD44 and COL6A2–(ITGA1+ITGB1) using SpaGene spatial interaction scores [37] demonstrated that the COL6A2–CD44 axis maintained higher levels in both core and border zones (Figure 5E, Table S10). For the multi-subunit complex, both ITGA1 and ITGB1 signals were higher in the border zone than in the core across a subset of samples (Table S10), consistent with single-cell predictions that active COL6A2-associated molecular interaction signals persist at the core–border interface. GAM fitting further revealed that macrophage M2 polarization scores progressively increased closer to the core zone (Figure 5F), while transcriptomic heterogeneity was significantly higher (*p* = 0.0034) in the core than in the distal zone (Figure 5G).

To characterize the molecular features of the core zone, we performed inter-group differential analysis across 12 samples and identified consensually upregulated stromal genes, including *COL1A1*, *TGFBI*, and *FN1*, which were enriched in collagen fibril organization and extracellular matrix assembly pathways (Figure 5H,I). These findings suggested that the core zone constructs a local physical barrier through high expression of stromal molecules. In addition, CNV scores were higher in the core zone than in the distal zone, indicating greater genomic instability (Figure 5J, Table S10). In summary, the AEIC core zone represents a spatial structure characterized by matrix remodeling, immune exclusion, and genomic instability. Although NK_CD16− cell abundance was low in the core zone, COL6A2-mediated molecular interaction signals remained detectable at the core–border interface.

### 2.6. AEIC-Derived 16-Gene Prognostic Signature Stratifies Patient Survival in ccRCC

**Figure 6:**
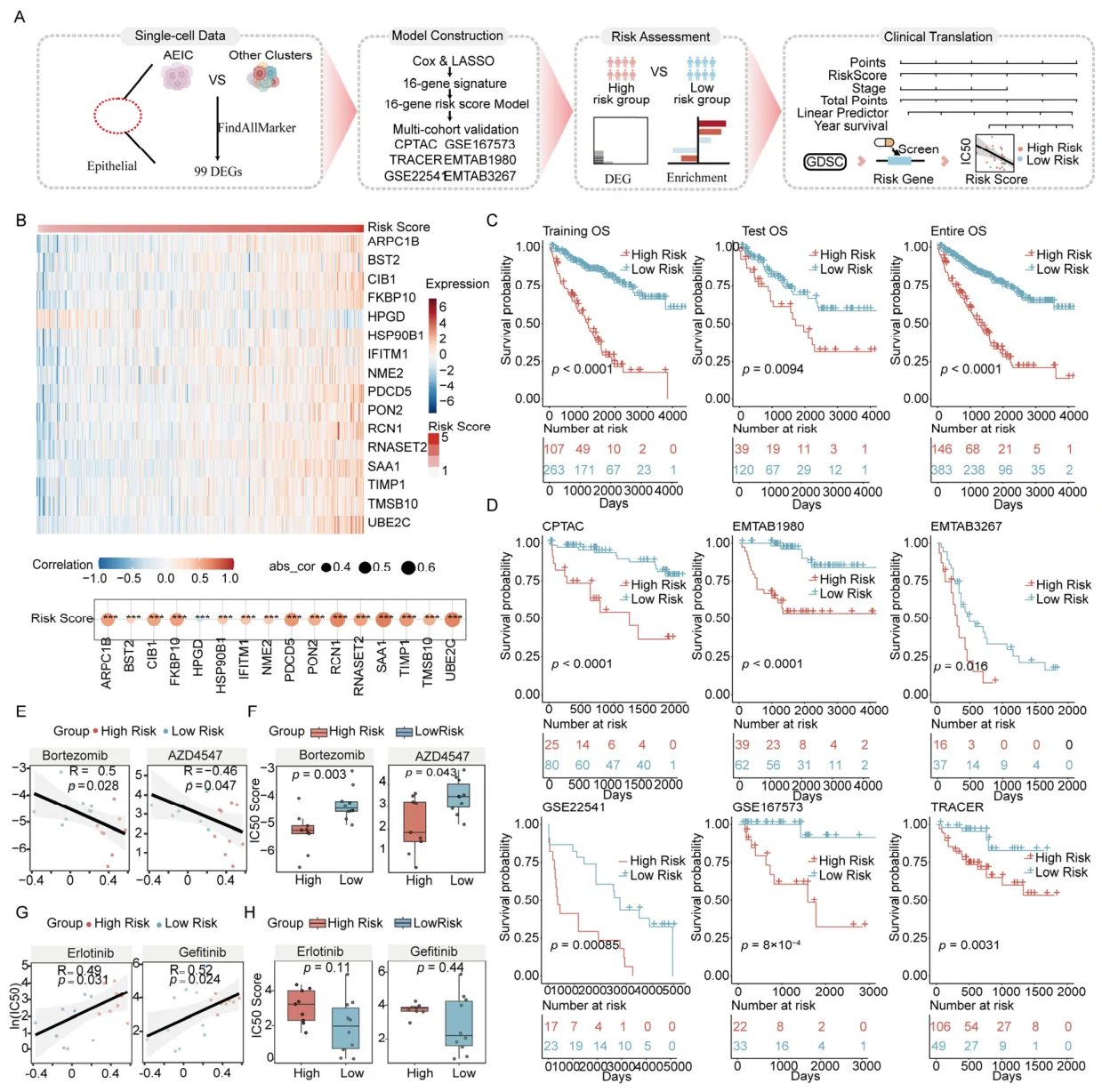
AEIC-derived 16-gene prognostic signature stratifies patient survival and is associated with drug sensitivity in ccRCC cell lines. (**A**) Flowchart of feature screening and model construction. (**B**) Expression patterns of the 16 genes comprising the risk score and correlation with the risk score (***, *p* < 0.001). (**C**) OS curves for the TCGA-KIRC training set, testing set, and entire cohort. (**D**) OS curves for external validation cohorts. (**E**) Correlation of risk scores with half maximal inhibitory concentration (IC50) values of Bortezomib and AZD4547. (**F**) Boxplot comparison of IC50 values of Bortezomib and AZD4547 between the high- and low-risk groups. (**G**) Correlation of risk scores with IC50 values of Erlotinib and Gefitinib. (**H**) Boxplot comparison of IC50 values of Erlotinib and Gefitinib between the high- and low-risk groups.

Given the established association between AEIC abundance and patient prognosis, we sought to develop a clinically applicable gene expression signature for prognostic stratification in ccRCC. Using differentially expressed genes between AEIC and other epithelial subclusters, we performed univariate Cox regression followed by LASSO-Cox regression analysis in the TCGA-KIRC training cohort, which identified a 16-gene prognostic signature (Figure 6A and Figure S10A, Section 4). *HPGD* was identified as the sole protective gene, whereas the remaining 15 genes, including *ARPC1B* and *BST2*, were risk genes with significant positive correlations with the risk scores (Figure 6B and Figure S10B). Univariate Cox regression analyses of all 16 genes were performed in the TCGA-KIRC cohort and six independent external cohorts (Table S11). Although gene-level significance varied, several genes showed consistent hazard ratio directions across multiple external cohorts. High-risk patients exhibited significantly poorer OS across the TCGA-KIRC training, test, and entire cohorts (Figure 6C), and the prognostic association of the 16-gene risk score was further validated in all six independent external cohorts (all *p* < 0.05; Figure 6D and Table S11), supporting the reproducibility of the 16-gene signature.

To explore the biological differences underlying this prognostic stratification, we compared differentially expressed genes between the high- and low-risk groups. Genes upregulated in the high-risk group were significantly enriched in immunosuppressive pathways, including IL-17 and TGF-β signaling, whereas genes upregulated in the low-risk group were mainly associated with renal metabolic pathways, including butyrate metabolism and PPAR signaling (Figure S10C,D). Univariate and multivariate Cox regression analyses confirmed that the risk score remained an independent prognostic factor after adjustment for age and stage (univariate HR = 3.968, multivariate HR = 2.627, *p* < 0.0001, Figure S10E). An integrated nomogram incorporating the risk score and clinical factors showed that the risk score contributed the most points to the nomogram (Figure S10F). Pathway enrichment analysis of the 16 signature genes identified five significant pathways mainly related to angiogenesis and vascular remodeling, cell-cycle regulation, and PI3K-related signaling (Figure S10G). These findings provided additional biological context for the association of the AEIC-related risk phenotype with aggressive tumor characteristics and therapeutic response.

As an application of the AEIC-derived prognostic signature, we explored whether the risk phenotype was associated with drug-sensitivity patterns in ccRCC cell lines using the GDSC2 database (Materials and Methods). Because only 12 of the 16 signature genes were represented in ccRCC cell lines, we reconstructed a 12-gene risk score. This score correlated strongly with the original 16-gene score in TCGA-KIRC (Pearson r = 0.87, *p* < 0.001), retained 83.77% of high-risk and 83.71% of low-risk classifications, and significantly stratified overall survival (Figure S11A,B). Eight compounds showed significant negative correlations between IC50 values and the risk score (|r| > 0.5, *p* < 0.05), with significantly lower IC50 values in high-risk cell lines (Figure 6E,F and Figure S11C,D). Among these, the proteasome inhibitor Bortezomib [38] and the FGFR inhibitor AZD4547 [39] showed the strongest negative correlations. In contrast, the EGFR inhibitors Erlotinib [40] and Gefitinib [41] showed positive correlations and higher IC50 values in high-risk cell lines (Figure 6G,H). We also analyzed Axitinib [42,43] and Temsirolimus [44], two established ccRCC therapies, which were available in GDSC2 but not included in the initial screen. Neither showed a significant correlation with the risk score or a significant difference in IC50 between risk groups (Figure S11E,F).

## 3. Discussion

Profound intra-tumoral heterogeneity is a hallmark of ccRCC [37,45] and contributes to uneven immune cell distribution and spatial immune exclusion, thereby limiting the efficacy of immunotherapy and leading to poor clinical outcomes [16,46]. However, the malignant subpopulations responsible for establishing this immune-excluded architecture remain poorly defined [13,47]. By integrating single-cell transcriptomics, matched scRNA-seq and scATAC-seq, spatial transcriptomics, and bulk RNA-seq cohorts, we identified AEIC as a core malignant subpopulation that orchestrates spatial immune exclusion in ccRCC. AEIC is characterized by high genomic instability and stemness, and is associated with adverse clinical outcomes, and its derived 16-gene signature provides a practical framework for patient prognostic stratification and further exploration of the biological and therapeutic features associated with the AEIC phenotype.

The aggressive phenotype of AEIC is likely associated with its genomic instability and distinct epigenetic landscape. The high CNV burden and elevated stemness observed in AEIC suggest that this malignant subpopulation has acquired a genomically unstable yet highly adaptable state, which may confer a selective survival advantage during ccRCC progression [48]. *MAZ* and *RUNX1* ranked highest in our integrative multi-omics screening and were identified as the core regulators of the AEIC transcriptional program. Given their complementary biological functions, these two TFs may cooperate to establish the regulatory landscape of AEIC. *MAZ* has been implicated in tumor proliferation and metastasis [49,50], whereas *RUNX1* promotes EMT and inflammatory transcriptional programs [51]. Among the downstream targets, TGFBI was particularly notable for its increased chromatin accessibility and elevated transcription in AEIC. As an extracellular matrix protein involved in stromal remodeling and immune regulation [51], TGFBI may link epigenetic reprogramming to the aggressive phenotype of AEIC. In contrast, the reduced chromatin accessibility of the canonical transcriptional repressor BCL11A suggests that the AEIC epigenetic landscape may involve both transcriptional activation and derepression. These findings suggest that coordinated activation of *MAZ/RUNX1*-centered regulatory programs may contribute to the acquisition and maintenance of the aggressive phenotype of AEIC. The bulk ATAC-seq validation supported the *MAZ*–*COL6A2* axis, whereas *RUNX1*-targeting *TGFBI* loci did not show significant changes. This discrepancy may reflect dilution of AEIC+ cell-specific signals in bulk tissue, as well as the greater reliance of *RUNX1* on motif occupancy than on overall chromatin openness. The core regulatory loci showed H3K27ac overlap rates comparable to those of global dREs, supporting their representativeness. Notably, although the *COL6A2* locus did not reach the H3K27ac overlap threshold, its significantly increased ATAC-seq accessibility supports this regulatory relationship. Furthermore, a published ccRCC epigenome–transcriptome study [52] independently identified *RUNX1* as an overexpressed gene driven by active histone modifications and CpG hypomethylation, supporting its epigenetic activation in ccRCC. Although direct binding of *RUNX1* to the TGFBI enhancer awaits confirmation in kidney-derived cells, our findings highlight this regulatory axis as a promising target for future mechanistic studies.

Previous studies have suggested that stromal remodeling contributes to the formation of a physical barrier that mediates spatial immune exclusion in ccRCC [53]. Our spatial transcriptomic analysis extends this concept by identifying AEIC as a potential clonal origin of this barrier. The AEIC-rich core was characterized by abundant stromal components, including *COL1A1* and *FN1*, together with enrichment of extracellular matrix organization pathways. This barrier coincides with M2-like macrophage polarization, increased genomic instability, and progressive exclusion of NK_CD16− cells from the tumor core, suggesting that AEIC contributes to the formation of an immune-excluded niche. Our data indicate that AEIC actively communicates with neighboring immune cells through COL6A2-dependent signaling. Although NK_CD16− cells were largely excluded from the core region, spatial transcriptomics consistently detected COL6A2 and its receptors at the interface between the core and border zones, indicating that ligand–receptor interactions remain active despite spatial segregation. Previous studies have shown that COL6A2 interacts with ITGB1 to activate Wnt/β-catenin signaling and promote EMT progression [53]. Our findings extend this observation by suggesting that COL6A2 may also participate in shaping the immune microenvironment surrounding AEIC.

Accurate identification of patients with aggressive disease is essential for improving prognostic stratification in ccRCC. Unlike conventional prognostic models developed directly from bulk transcriptome data, our prognostic signature was derived from the AEIC malignant subpopulation identified through multi-omics single-cell analyses, and therefore reflects transcriptional features associated with aggressive tumor behavior. The robust performance of the 16-gene signature across multiple independent cohorts supports its prognostic relevance. Among the signature genes, *HPGD* was the only protective factor, consistent with its reported role in prostaglandin metabolism and immune regulation [54], whereas most of the remaining genes have been implicated in extracellular matrix remodeling, inflammation, and cell-cycle progression [55,56], which aligns with the AEIC phenotype. Pathway enrichment of the 16 signature genes highlighted angiogenesis and vascular remodeling, cell-cycle regulation, and PI3K-related signaling. Published studies have also linked individual signature genes to therapeutic response or resistance in ccRCC (Table S12). *SAA1* has been associated with resistance to anti-PD-1 therapy, and preclinical studies suggest that *SAA1* blockade can enhance the antitumor effects of anti-PD-1 treatment, including in combination with a TKI [57,58]. Similarly, *FKBP10* inhibition enhanced the antitumor effect of the HIF-2α inhibitor PT2385 in preclinical models [59]. These findings provide mechanistic context for the potential therapeutic relevance of individual signature genes but do not establish the 16-gene signature itself as a treatment-response biomarker.

Methodologically, our multi-omics integration strategy combined scRNA-seq, scATAC-seq, spatial transcriptomics, and bulk transcriptome cohorts to dissect AEIC from epigenetic regulation to spatial organization. The MCRS framework provided a systematic way to prioritize candidate TFs by integrating motif accessibility, cis-regulatory activity, and gene expression. The spatial domain model linked molecular alterations to tissue architecture and local immune organization. Together, these approaches connected transcriptional states with their regulatory programs and spatial contexts. Our integrative framework therefore provides a strategy for connecting epigenetic regulation to spatial organization and clinical translation through multi-layered data integration.

This study has several limitations. First, our conclusions are primarily derived from bioinformatic analyses and in situ spatial mapping, lacking functional validation experiments such as knockdown or overexpression of *MAZ*/*RUNX1* or *TGFBI*. Second, the scRNA-seq datasets integrated in this study were derived from different cohorts with potentially inconsistent sampling strategies, including variations in tumor location, tumor size, and the definition of adjacent normal tissues. Although we applied Harmony batch correction and observed consistent findings across datasets, residual heterogeneity may exist, warranting future studies with standardized sampling protocols. Third, the AEIC-driven stromal remodeling and immune exclusion identified here require orthogonal validation using multiplex immunofluorescence in independent clinical cohorts. Fourth, the inherent dropout phenomenon of single-cell and spatial technologies may obscure low-abundance transcripts and regulatory events. Fifth, the drug-sensitivity analysis was exploratory and based on GDSC2 cell lines, with only 12 of the 16 signature genes represented. The reconstructed 12-gene score does not fully reproduce the patient-derived signature. These drug-response associations should therefore be considered preliminary and require validation in functional models and clinical cohorts with treatment-response data. Despite these limitations, our study identifies AEIC as a critical malignant subpopulation that links epigenetic dysregulation to spatial immune exclusion in ccRCC and provides a framework for prognostic stratification and exploration of therapeutic vulnerabilities associated with the AEIC phenotype.

## 4. Materials and Methods

### 4.1. Data Collection

Four scRNA-seq datasets generated using the 10X Genomics platform were downloaded from the Gene Expression Omnibus (GEO, https://www.ncbi.nlm.nih.gov/geo/, accessed on 20 March 2024, GSE156632, GSE178481, GSE210038) and Li_R [15,21,60,61]. Following quality control, these datasets yielded 70 high-quality samples from 32 patients. A matched scRNA-seq and scATAC-seq dataset (GSE207493), consisting of 18 qualified samples from 18 patients [62], was incorporated for integrative transcriptomic and epigenomic analyses. Bulk ATAC-seq and H3K27ac ChIP-seq data from 11 paired normal–tumor samples in the independent cohort GSE309697 were used to validate dRE accessibility and enhancer activity. Spatial transcriptomic data were obtained from Zenodo (https://zenodo.org/record/8063124, accessed on 3 December 2025) [14]. The TCGA-KIRC cohort, downloaded from The Cancer Genome Atlas (TCGA, https://portal.gdc.cancer.gov/, accessed on 20 March 2024), was used as the discovery cohort. Five independent bulk transcriptome cohorts from CPTAC [63], BioStudies (E-MTAB-1980 and E-MTAB-3267) [64], GEO (GSE22541 and GSE167573) [65,66], and TRACERx [45] served as external validation cohorts. Drug sensitivity data, including half-maximal inhibitory concentration (IC50) values and matched transcriptomic profiles for 19 ccRCC cell lines across 288 compounds, were obtained from the GDSC2 database (https://www.cancerrxgene.org/, accessed on 13 January 2026).

### 4.2. Single-Cell Transcriptomic Data Processing

Four independent scRNA-seq datasets were processed using the Seurat (v4.4.0) package [67]. Samples with mixed typologies, atypical pathological features, metastatic lesions, tumor thrombi, or prior neoadjuvant therapy were excluded based on clinicopathological metadata. Cell-level QC was performed according to the criteria established in the original publications of the respective datasets [15,21,60,61], with dataset-specific preprocessing strategies applied using Seurat. For GSE156632 and GSE210038, low-quality cells and doublets were removed based on detected gene numbers, mitochondrial content, and the DoubletFinder (v2.0.6) R package [68]. An additional upper threshold for detected genes was applied to GSE156632 beyond the original criteria to reduce potential technical artifacts. For GSE178481 and Li_R, erythroid lineage cells and cells derived from non-target regions were excluded using published cell annotations from the original studies. Subsequently, protein-coding genes were retained based on GENCODE (GRCh38) annotations, and mitochondrial genes, ribosomal genes, and samples with fewer than 1000 cells were removed to construct the final integrated matrix. The integrated data then underwent normalization and log-transformation, followed by selection of the top 3000 highly variable genes (HVGs) for principal component analysis (PCA). Batch effects across cohorts were corrected using the harmony algorithm [28], and UMAP was performed based on the corrected embeddings for visualization.

### 4.3. Cell Annotation

Cell clusters were annotated based on canonical marker genes identified through differential expression analysis using the Wilcoxon rank-sum test and further validated by cross-cohort Pearson correlation analysis. Major non-epithelial lineages were subsequently subjected to lineage-specific sub-clustering to improve annotation resolution. Immune cell subtypes, including T/NK and myeloid populations, were annotated using CellTypist [28] and further refined based on differential expression analysis and known marker genes. To validate the annotations of T-cell subsets, SCENIC was performed using the SCENIC (v1.2.2) package [30] to infer TF regulatory activity. Endothelial and stromal populations were annotated based on differentially expressed genes and relevant published literature [15,60,61].

### 4.4. Malignant Cell Identification and AEIC Signature Construction

Malignant epithelial cells were identified based on epithelial lineage annotation together with inferred chromosomal CNV profiles. The epithelial subpopulation with concurrent activation of EMT and inflammatory signaling pathways was designated as the aggressive EMT-inflammatory clone (AEIC). Genome-wide CNVs were inferred using the inferCNV (v1.22.0) package [31] with normal proximal tubule cells (ref-Normal_PT) as the diploid reference (cutoff = 0.1) [69]. The inferred CNV matrix was Z-score-normalized, linearly rescaled to a range of −1 to 1, and summarized as a cumulative CNV score by calculating the quadratic sum across all genes. Differences in CNV scores among epithelial subpopulations were evaluated using a two-tailed Wilcoxon rank-sum test with ref-Normal_PT cells as the reference.

To prioritize genes with high specificity for AEIC, we defined a specificity ratio (SR):

(1) $$SR_{g}=\frac{P_{\mathrm{target}}+0.01}{P_{\mathrm{background}}+0.01}$$

where *P*_target_ and *P*_background_ represent the detection proportions of gene g in the AEIC and remaining epithelial subpopulations, respectively, and 0.01 was included as a smoothing constant to ensure numerical stability. Genes with high SR values were iteratively filtered based on UCell scoring [70], yielding a 43-gene AEIC signature. The robustness of the AEIC signature was assessed by 1000 bootstrap iterations. The relative abundance of AEIC in bulk transcriptome cohorts was estimated using ssGSEA implemented in the GSVA (v2.4.9) package [71], and the resulting enrichment scores were used for downstream prognostic analyses and risk score calculation.

### 4.5. Assessing Correspondence with Established Molecular Subtypes

To evaluate the correspondence between AEIC and established ccRCC molecular subtypes, a hypergeometric test was performed to assess the overlap between the 43-gene AEIC signature and published marker gene sets of the consensus molecular subtypes derived from the GSE210038 cohort.

### 4.6. Matched scRNA-seq and scATAC-seq Integration

Matched scRNA-seq and scATAC-seq data (GSE207493) were integrated using the ArchR (v1.0.2) package [72]. We constructed tile matrices using 500-bp tiles from the scATAC-seq, with quality control thresholds set to a unique fragment count of 1000–20,000 and a TSS enrichment score > 8. RCC96 was excluded because of an insufficient number of high-quality matched cells. AEIC labels were first assigned in the matched scRNA-seq dataset. After normalization and batch correction, each malignant epithelial cell was scored using the 43-gene signature with the UCell algorithm. Cells in the top and bottom 25% of the score distribution were defined as AEIC+ and AEIC−, respectively. These labels were transferred from the scRNA-seq dataset to the matched scATAC-seq dataset using the addGeneIntegrationMatrix function, with cells retained when the predictedScore exceeded 0.5.

### 4.7. Identification of AEIC+-Specific dREs

To identify high-confidence CREs, correlations between chromatin accessibility peaks and nearby gene expression (within 250 kb) were calculated using ArchR’s addPeak2GeneLinks function (FDR < 0.01) [73]. The *Tau* index was applied to evaluate cell-type specificity of regulatory elements [74]. To focus on tumor-specific regulatory elements, distal peaks specifically accessible in AEIC+ cells were compared with ENCODE reference data from normal kidney tissue. The resulting tumor-specific dREs were retained for subsequent transcription factor enrichment analysis.

### 4.8. Core Transcription Factor Screening

To identify the upstream core TFs driving AEIC+ dREs, an integrative screening strategy was applied. Regulatory regions were first annotated using the CIS-BP database [75] via the addMotifAnnotations function in the ArchR package. Motif accessibility deviations between AEIC+ and AEIC− cells were then quantified using the addDeviationsMatrix function. Candidate TFs supported by both motif annotation and accessibility analyses were further prioritized using a multi-omics joint regulatory score (MCRS), which integrates cis-regulatory flux, motif enrichment, and transcriptional activity. The score was calculated as follows:

(2) $$\mathrm{MCRS}\left(TF_{j}\right)=\mathrm{norm}\left(\sum_{i\in P_{j}}{\mathrm{Cor}}_{i}\right)+\mathrm{norm}\left(E_{j}\right)+\mathrm{norm}\left(\mathrm{LF}C_{j}\right)$$

where MCRS(TF*_j_*) represents the comprehensive score of transcription factor j. The individual sub-metrics are defined as follows:

1. Cis-regulatory flux ($\sum_{i\in p_{j}}{\mathrm{Cor}}_{i}$): the sum of P2G correlation coefficients associated with all AEIC+-specific dREs bound by TF*_j_*.
2. Motif enrichment intensity (*E_j_*): the ratio of the relative distribution frequencies of the TF motif in the characteristic regions versus the genomic background regions, correcting for background distribution bias:
  (3) $$E_{j}=\frac{f_{\mathrm{marker},j}}{f_{\mathrm{background},j}}=\frac{N_{\mathrm{marker},j}/\sum N_{\mathrm{marker}}}{N_{\mathrm{total},j}/\sum N_{\mathrm{total}}}$$
  where *N*_marker,*j*_ and $\sum N_{\mathrm{marker}}$ represent the number of motifs for transcription factor *j* identified within the characteristic peak regions of the AEIC+ subpopulation (i.e., dREs associated with AEIC marker genes) and the total number of all transcription factor motifs within these regions, respectively. *N*_total,*j*_ and $\sum N_{\mathrm{total}}$ represent the number of motifs for transcription factor *j* identified within the open chromatin regions of both the AEIC+ and AEIC- subpopulations (the background set) and the total number of all transcription factor motifs within this background set, respectively.
3. Transcriptional activity (LFC*_j_*): The avg_log2FC value extracted from differential expression analysis between AEIC+ and AEIC− subpopulations via the Wilcoxon rank-sum test using the FindMarkers function in Seurat package (*p*_adj < 0.05, avg_log2FC > 0).

A multi-step convergence strategy was applied to sequentially filter candidates through these dimensions, and genomic footprinting analysis was performed using the getFootprints function to validate the actual binding strength of the TFs from the perspective of physical protein occupancy. Finally, genomic footprinting analysis was performed to validate the identified core TFs in AEIC+ cells.

### 4.9. Analysis of dRE Accessibility and Enhancer Activity in an Independent Cohort

The 442 dREs and core TF–target gene enhancer regions were mapped to genomic coordinates and compared with H3K27ac ChIP-seq peaks from tumor samples in the independent cohort GSE309697. Overlapping regions were identified using genomic interval overlap analysis, and the proportions of H3K27ac-positive regions among the core enhancers and all 442 dREs were compared using Fisher’ s exact test. A 1000-iteration genomic permutation test was performed to assess whether the observed overlap exceeded random expectation. To assess differential chromatin accessibility, normalized ATAC-seq signals were extracted from BigWig files for each dRE across 11 paired tumor–normal samples. Paired Wilcoxon signed-rank tests were performed for each dRE, and fold changes were calculated as the log2 ratio of mean tumor to mean normal signal. Regions with *p* < 0.05 and positive or negative log2FC were classified as showing increased or decreased accessibility, respectively. Core TF–target gene regulatory axes were further examined individually to assess their accessibility differences between tumor and normal tissues.

### 4.10. Cell–Cell Communication Analysis

The AEIC subpopulation, other tumor-enriched malignant epithelial populations (Ro/e > 2), and eleven non-epithelial subpopulations significantly enriched in tumor tissues (Ro/e > 1) were selected for communication analysis. A cell co-occurrence network was constructed using Pearson correlation coefficients calculated from subpopulation abundances across samples. Cell–cell communication probabilities at both the interaction and pathway levels were inferred using the CellChat (v2.2.0) package [34] with the CellChatDB.human database as the ligand–receptor reference. Total outgoing signaling activities were compared between AEIC and other tumor cells. To quantify pathway specific activation, an enrichment score was defined as:

(4) $$\mathrm{ES}=\frac{P_{\mathrm{AEIC}+}+\epsilon }{P_{\mathrm{Other tumor}}+\epsilon }$$

where *P*_AEIC+_ and *P*_Other tumor_ represent the cumulative communication probabilities of a given pathway in AEIC and other tumor-enriched malignant epithelial cells, respectively, and ϵ = 1 × 10^−3^ is a correction factor to smooth low background signals. The NicheNet algorithm [35] was further applied to predict ligand-driven transcriptional regulation in recipient cells and identify key ligand–target regulatory axes.

### 4.11. Spatial Slice Cell-Type Composition and Domain Modeling

Spatial deconvolution was performed using Cell2location [76] method to map single-cell reference signatures onto 12 Visium sections via a negative binomial regression model. Regions with the top 5% estimated AEIC abundance were defined as the tumor core. We then calculated the Euclidean distance d of each spot across the tissue section relative to this centroid. To systematically map the spatial gradient of the tumor microenvironment from the center to the periphery, we stratified the tissue into three concentric functional zones based on the quantiles of d: the Core zone (d ≤ Q10, capturing the primary cluster of high-abundance AEIC), the Border zone (Q10 < d ≤ Q30), and the Distal zone (d > Q30).

To identify cell populations consistently associated with the AEIC core, cell spatial distribution rankings across samples were integrated using the RRA algorithm [36]. For each section, spots in the top 20% proportion for each cell type were extracted, and the average spatial distance from these spots to the starting boundary of the AEIC core was calculated to generate a ranked list. UCell scoring combined with generalized additive model (GAM) was used to characterize the spatial trajectory of M2 macrophage polarization gene sets along the distance gradient [77,78]. Differential expression analysis between the Core and Distal zones (log2FC > 0.2, adjusted *p* < 0.05) identified consensus stromal genes that were upregulated in more than 50% of samples, followed by Gene Ontology (GO) enrichment analysis. We also inferred chromosomal copy number variations in each zone using the SPATA2 (v3.1.4) package [79] and calculated CNV scores to assess regional genomic instability.

### 4.12. Generation of an AEIC-Derived 16-Gene Prognostic Signature

To develop a clinically applicable prognostic signature, we used the TCGA-KIRC cohort as the discovery dataset. The cohort was randomly divided into a training set (70%) and a test set (30%), while the entire cohort served as the internal validation set. Six independent external transcriptomic cohorts were included for external validation. Based on differentially expressed genes between AEIC and the remaining epithelial subpopulations, we first performed univariate Cox regression proportional hazards regression analysis to screen prognosis-associated genes (*p* < 0.05), followed by LASSO-Cox regression analysis with 10-fold cross-validation to eliminate multicollinearity and construct a 16-gene prognostic signature. Risk scores were calculated as the weighted sum of gene expression values using the LASSO-derived coefficients, and the surv_cutpoint function in the survminer package was used to divide patients into high- and low-risk groups. We evaluated overall survival (OS) using Kaplan–Meier curves with the log-rank test in the training, test, and internal validation cohorts, and further validated the model in six external cohorts. Univariate and multivariate Cox regression analyses were performed to determine whether the risk score was an independent prognostic factor after adjustment for age, stage, and grade. A prognostic nomogram integrating the risk score and clinical variables was constructed using the rms package, and time-dependent ROC analysis was used to evaluate predictive accuracy.

### 4.13. Exploratory Drug Sensitivity Analysis

To explore potential therapeutic vulnerabilities associated with the AEIC-related risk phenotype, gene expression profiles and IC50 values for ccRCC cell lines were obtained from the GDSC2 database. We first performed Pearson correlation analysis (|r| > 0.5 and *p* < 0.05) to identify candidate drugs whose sensitivity was significantly associated with risk gene expression. The risk score for each cell line was calculated using the available 16 signature genes and the LASSO-derived coefficients. Because only 12 of the 16 signature genes were represented in the GDSC2 expression matrix, the cell-line risk score was reconstructed using these 12 genes. The concordance between the 12- and 16-gene risk scores was evaluated in the TCGA-KIRC cohort. Specifically, we calculated the Pearson correlation between the two risk scores, compared the retention of high- and low-risk classifications based on the original 16-gene score, and performed Kaplan–Meier survival analysis with the log-rank test to assess whether the reconstructed score stratified overall survival. The median reconstructed risk score was used to stratify cell lines into high- and low-risk groups. Differences in drug sensitivity between the two groups were evaluated using the Wilcoxon rank-sum test based on IC50 values. Compounds showing significant associations with the risk score or differential sensitivity between risk groups were considered candidate therapeutic agents for further investigation.

### 4.14. Functional Enrichment Analysis

To characterize the biological functions associated with epigenetic, communication, and spatial analyses, multiple functional enrichment approaches were applied. For epigenetic analyses, biological pathways associated with AEIC-specific dREs were identified using the rGREAT (v2.13.1) package [80]. For cell–cell communication, pathway-specific signaling activity was quantified by calculating the communication probability-based ES between cell groups. For spatial transcriptomic analysis, macrophage M2 polarization was quantified using the UCell algorithm based on canonical polarization gene sets. Functional enrichment of spatial core-derived consensus genes and differentially expressed genes associated with the prognostic signature was performed using GO and Kyoto Encyclopedia of Genes and Genomes (KEGG) analyses implemented in the clusterProfile (v4.18.4) package [81], with *p* < 0.05 considered statistically significant. Functional enrichment analysis of the 16 genes comprising the prognostic signature was also performed using GO, KEGG, and Hallmark gene sets, with *p* < 0.05 considered statistically significant.

### 4.15. Statistical Analysis

All statistical analyses were performed using R. Differences between two groups were evaluated using the Wilcoxon rank-sum test, whereas comparisons among multiple groups were performed using the Kruskal–Wallis test. Gene set overlaps were assessed using the hypergeometric distribution test. Survival analyses were performed using Kaplan–Meier curves with log-rank tests, and univariate and multivariate Cox proportional hazards regression models were applied to identify independent prognostic factors. Pearson correlation analysis was used to evaluate associations between variables. Statistical significance was defined as: * *p* < 0.05, ** *p* < 0.01, *** *p* < 0.001; ns, not significant.

## 5. Conclusions

Our study identified AEIC as a distinct malignant epithelial subpopulation that co-activates EMT and inflammatory pathways in ccRCC. Through integrative single-cell and spatial multi-omics analyses, we showed that AEIC is associated with aggressive tumor behavior, spatial immune exclusion, and poor clinical outcome. We further established an AEIC-derived 16-gene prognostic signature with robust performance across multicenter cohorts and identified distinct drug-sensitivity patterns associated with the AEIC-related risk phenotype in ccRCC cell lines. Collectively, these findings advance our understanding of ccRCC heterogeneity and support the prognostic value of the AEIC-derived signature while highlighting potential therapeutic vulnerabilities that warrant further validation.

## Data Availability

The datasets presented in this study are publicly available data. No new data were generated for this research. Detailed accession numbers and cohort characteristics are provided in Table S8. The code generated or used during the study is available at GitHub (https://github.com/lanrifang1949/AEICanalysis, accessed on 13 January 2026). All plotting scripts were developed and executed using R version 4.3.1.

## Supplementary Materials

The following supporting information can be downloaded at: https://www.mdpi.com/article/10.3390/ijms27219668/s1.

## Abbreviations

ccRCC	Clear Cell Renal Cell Carcinoma
EMT	Epithelial–Mesenchymal Transition
AEIC	Aggressive EMT-Inflammatory Clone
ITH	Intra-Tumoral Heterogeneity
scATAC-seq	Single-Cell Assay for Transposase-Accessible Chromatin Using Sequencing
scRNA-seq	single-Cell RNA Sequencing
mCAF	Myofibroblastic Cancer-Associated Fibroblast
CapEC	Capillary Endothelial Cell
TipEC	Tip Endothelial Cell
CNV	Copy Number Variation
ssGSEA	Single-Sample Gene Set Enrichment Analysis
dREs	Distal Regulatory Elements
TFs	Transcription Factors
RRA	Robust Rank Aggregation
Mono_NC	Non-Classical Monocytes
IC50	Half Maximal Inhibitory Concentration
GEO	Gene Expression Omnibus
TCGA	The Cancer Genome Atlas
GDSC2	Genomics of Drug Sensitivity in Cancer 2
