# Supplementary material for: An Epigenetically Remodeled EMT–Inflammatory Clone Associated with Spatial Immune Exclusion in Clear Cell Renal Cell Carcinoma
Source: Int J Mol Sci. 2026 Oct 28;27(21):9668. doi: 10.3390/ijms27219668 (PMC13763239; doi:10.3390/ijms27219668)
Supplement: Supplementary file 1 [file ijms-27-09668-s001.zip › Supplymental Figure.pdf]

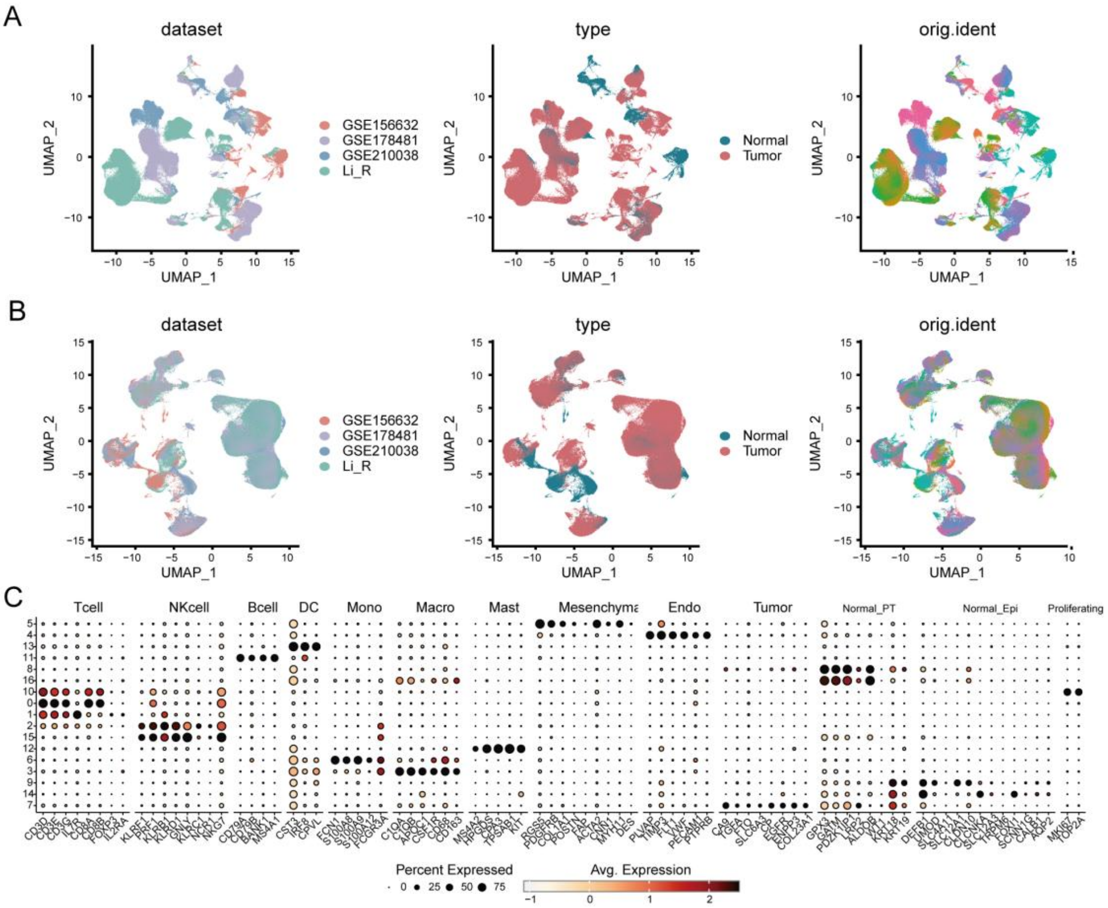

Figure S1. Single-cell RNA-sequencing data integration and cell lineage identification. (A) UMAP plots before Harmony batch correction colored by dataset, type, and sample. **(B)** UMAP plots after Harmony batch correction colored by dataset, type, and sample. **(C)** Bubble plot showing the expression of canonical marker genes across identified cell clusters. Dot size represents the percentage of expressing cells, and color intensity represents the average expression level.

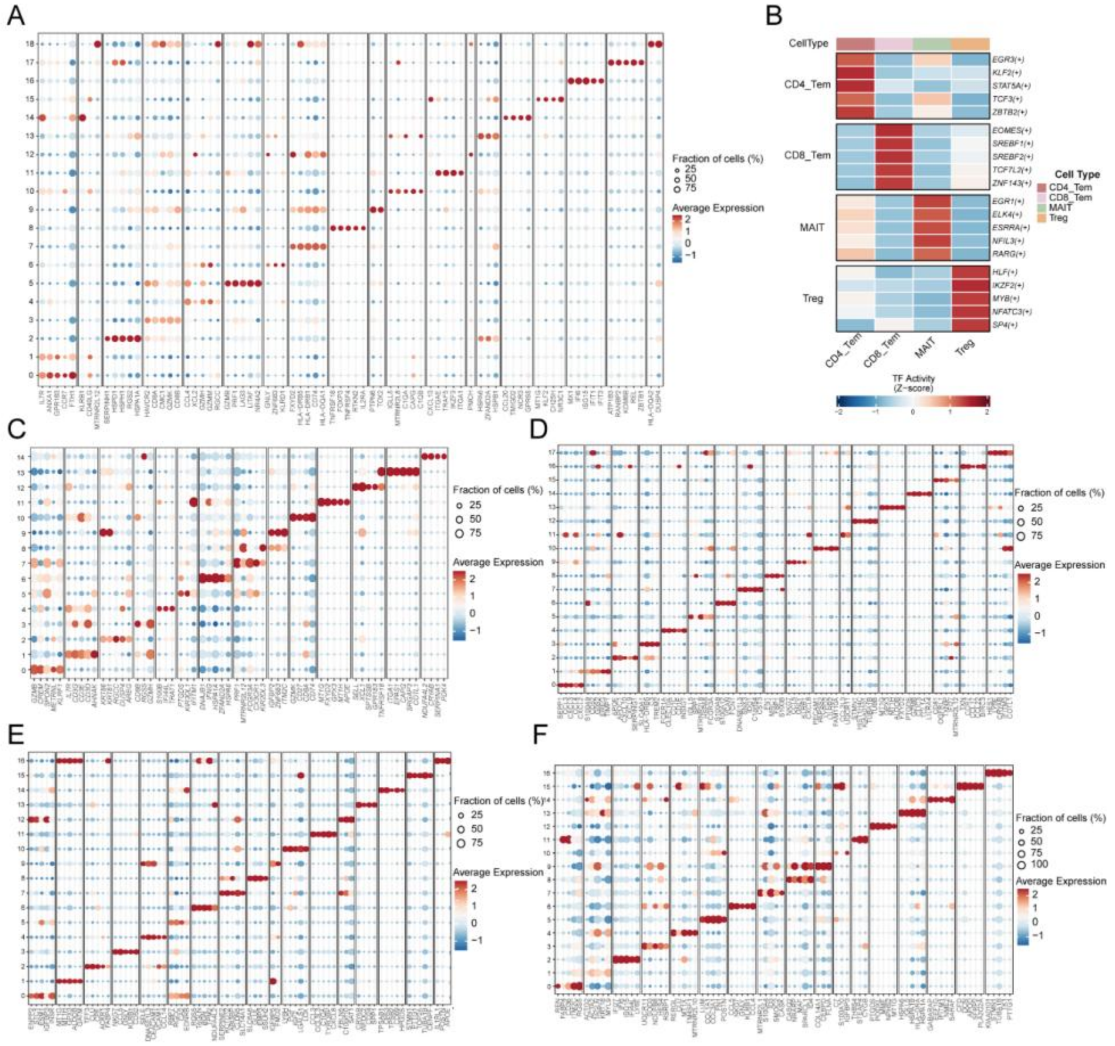

Figure S2. Marker gene expression and transcription factor activities across non-epithelial cell sub-lineages. (A) Bubble plot showing the differentially expressed genes across T-cell subclusters. **(B)** Heatmap showing the regulon activities of the top 5 transcription factors for each T cell subtype inferred by SCENIC analysis. **(C)** Bubble plot showing the differentially expressed genes across NK cell subclusters. **(D)** Bubble plot showing the differentially expressed genes across myeloid cell subclusters. **(E)** Bubble plot showing the differentially expressed genes across endothelial cell subclusters. **(F)** Bubble plot showing the differentially expressed genes across mesenchymal cell subclusters. Dot size represents the percentage of expressing cells, and color intensity represents the average expression level in (A), (C), (D), (E), (F).

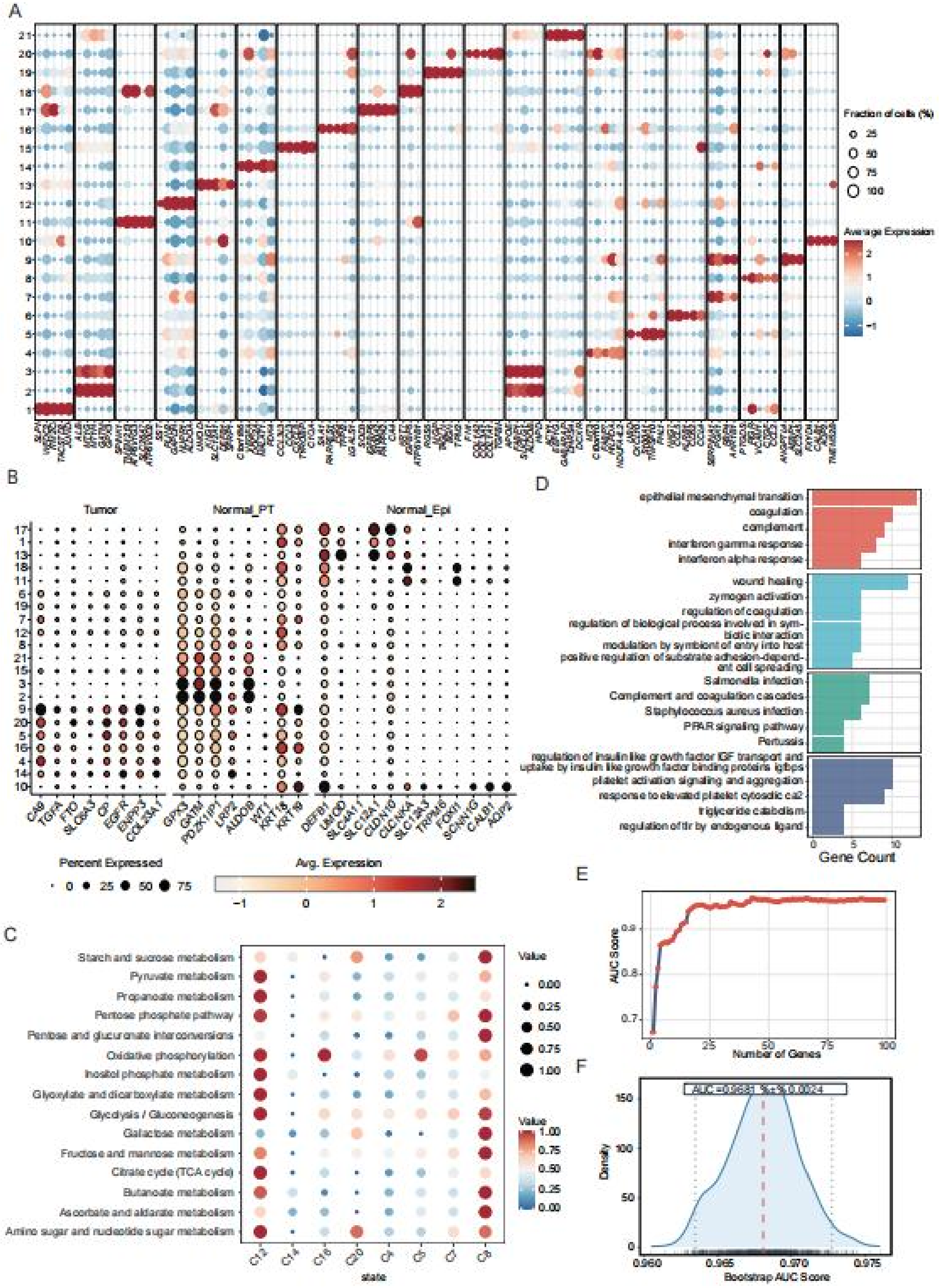

Figure S3. Characterization and functional annotation of the AEIC epithelial subcluster. (A) Bubble plot showing the differentially expressed genes across epithelial cell subclusters. **(B)** Bubble plot showing the expression of canonical ccRCC tumor markers, normal proximal tubule (PT) markers, and normal epithelial markers across epithelial subclusters. **(C)** Activity differences

of epithelial subclusters across canonical metabolic pathways. **(D)** Functional enrichment of differentially expressed genes in cluster 16 (AEIC). **(E)** Line plot showing the AUC scores as a function of the number of feature genes prioritized by expression specificity ratios. **(F)** Density plot showing the distribution of discriminant AUC scores validated across 1,000 bootstrap samplings.

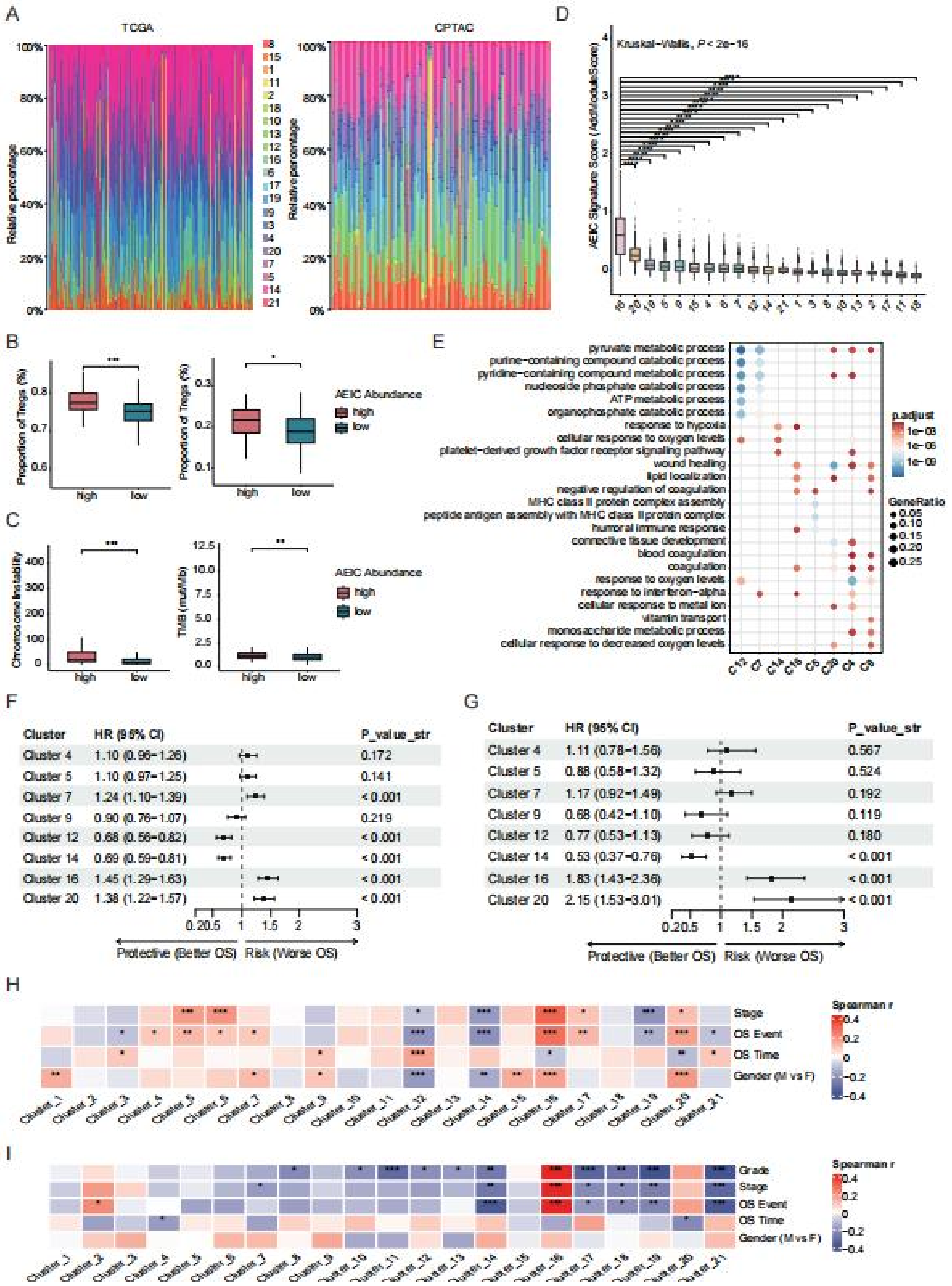

Figure S4. Epithelial subcluster abundance, clinical correlates, and prognostic significance of the AEIC. (A) Stacked bar plots showing the relative percentage distribution of different epithelial cell clusters within each sample across the TCGA and CPTAC cohorts estimated by BayesPrism deconvolution. **(B)** Treg infiltration between AEIC high- and low-abundance groups in the TCGA and CPTAC cohorts. **(C)** Box plots comparing chromosome instability scores (left, $P < 0.001$) and tumor mutation burden (TMB) scores (right, $P < 0.01$) between AEIC high- and

low-abundance groups in the TCGA cohort. **(D)** Box plots showing AEIC signature scores across all epithelial subclusters. **(E)** Bubble plot showing pathway enrichment of tumor-enriched epithelial subclusters. **(F – G)** Univariate Cox regression of tumor-enriched epithelial subcluster abundance in the TCGA **(F)** and CPTAC **(G)** cohorts. **(H – I)** Spearman rank correlation analysis between deconvoluted subcluster abundances and clinical parameters in TCGA-KIRC **(H)** and CPTAC-ccRCC **(I)** cohorts.

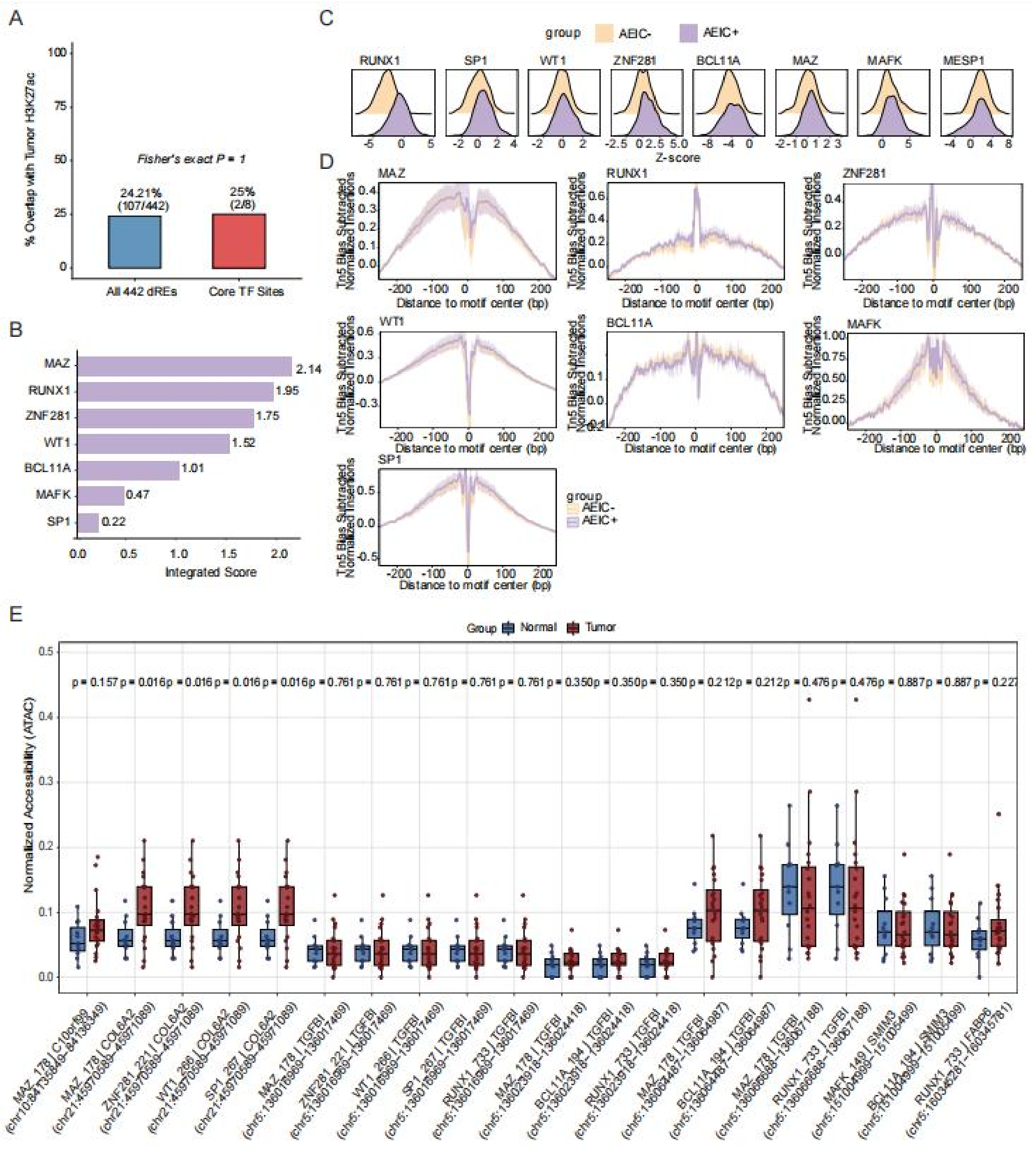

Figure S5. Regulatory potential and validation of epigenetic features of core transcription factors in the AEIC subpopulation. (A) Comparison of H3K27ac overlap rates between 8 core TF-targeted enhancer regions (Core) and 442 global distal regulatory elements. **(B)** Bar plot showing the integrated regulatory scores of the seven core transcription factors. **(C)** Density plots comparing the regulatory activity distribution of core transcription factors between the AEIC+ and AEIC- subpopulations. **(D)** Transcription factor footprinting analysis validating the genomic binding intensities of core transcription factors between the AEIC+ and AEIC- groups. **(E)** Bar plot comparing chromatin accessibility at core TF-enhancer sites between tumor and normal tissues in the GSE309697 cohort.

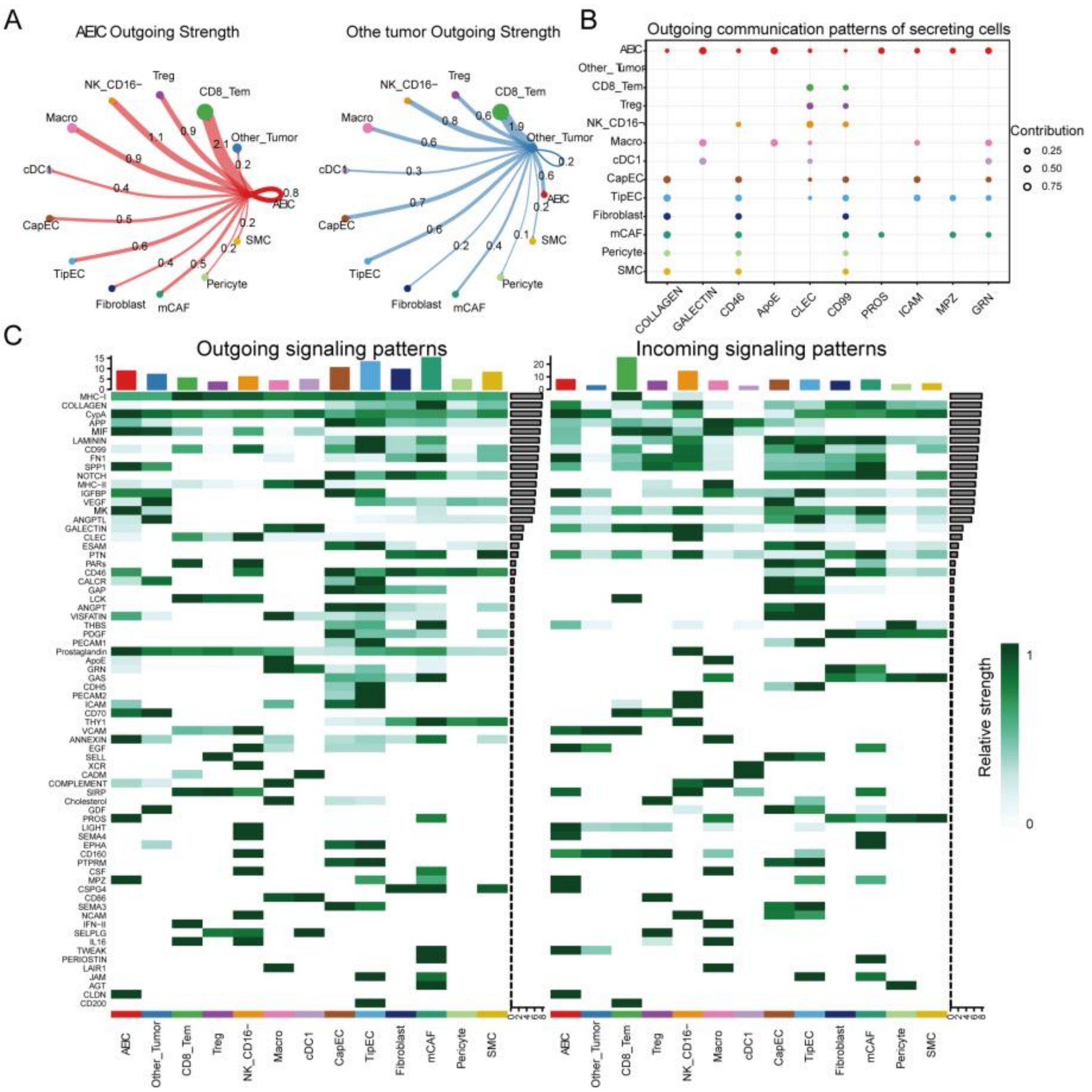

Figure S6. Signaling pathways in AEIC-mediated intercellular communication. (A) Network plots comparing the total outgoing signaling strengths between the AEIC subpopulation and other malignant epithelial subpopulations across microenvironmental cell types. **(B)** Dot plot showing the relative contribution of each cell subpopulation to specific outgoing communication pathways. Dot size represents the contribution value. **(C)** Heatmaps showing the global incoming and outgoing signaling patterns across all cell subpopulations, illustrating the relative strength of specific communication pathways (rows) for each cell type (columns).

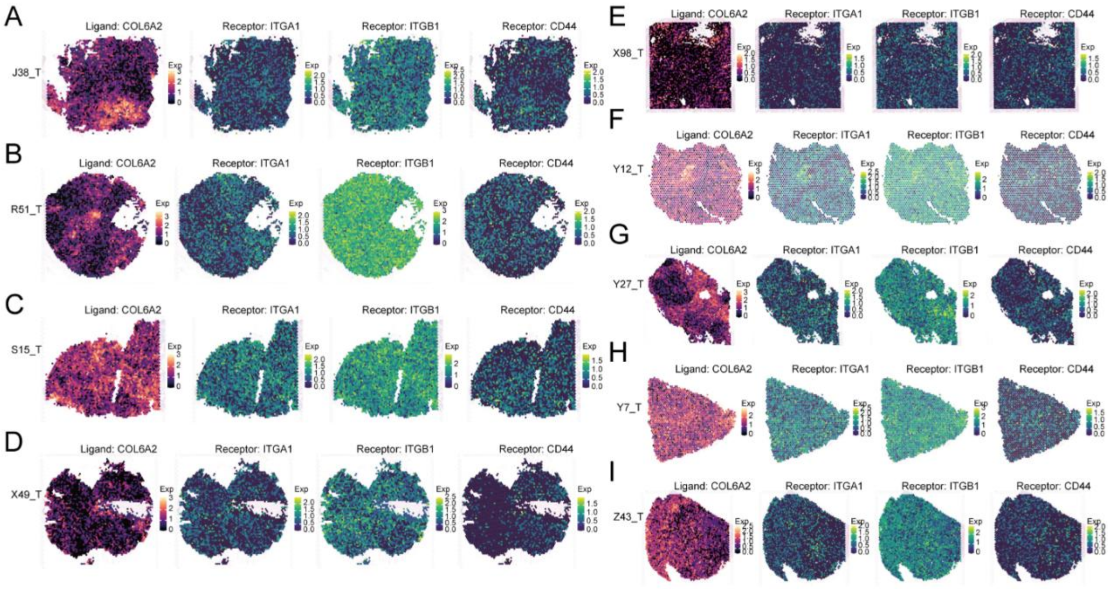

Figure S7. Spatial expression of key ligand and receptor genes across independent spatial transcriptomic sections. (A–I) Spatial expression heatmaps showing the distribution and expression levels of ligand *COL6A2* and receptors *ITGA1*, *ITGB1*, and *CD44* across nine independent Visium spatial transcriptomic tissue sections, including J38_T (A), R51_T (B), S15_T (C), X49_T (D), X98_T (E), Y12_T (F), Y27_T (G), Y7_T (H), and Z43_T (I). Color bars represent normalized expression levels.

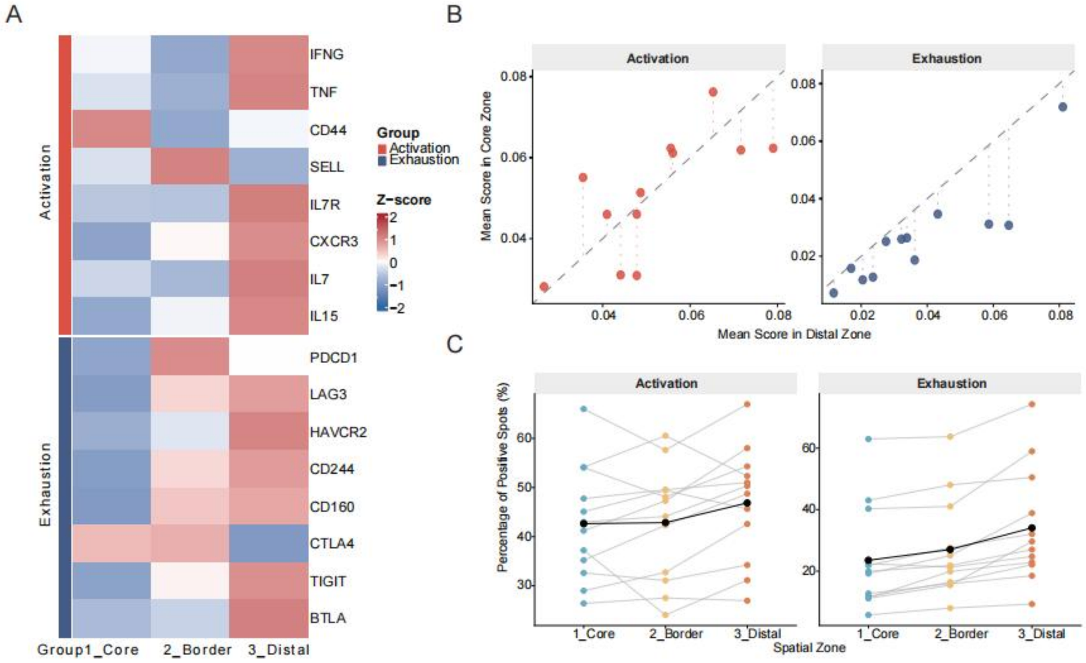

Figure S8. Spatial distribution and module scores of immune effector marker expression across tumor microenvironment regions. (A) Consensus heatmap showing Z-score normalized mean expression levels of 16 immune effector markers (activation and exhaustion) across Core, Border, and Distal regions (aggregated by median values across n = 12 spatial transcriptomics samples). **(B)** Scatter plots comparing the mean UCell module scores (mean_score) for T-cell activation (left) and exhaustion (right) between the core and distal regions across individual spatial transcriptomics samples. The dashed line represents the y = x identity line. **(C)** Line plots showing the percentage of positive spots (pos_pct, score > 0) for activation (left) and exhaustion (right) modules across core (1_Core), border (2_Border), and distal (3_Distal) regions in individual samples. Black connected dots represent the mean percentages across all samples.

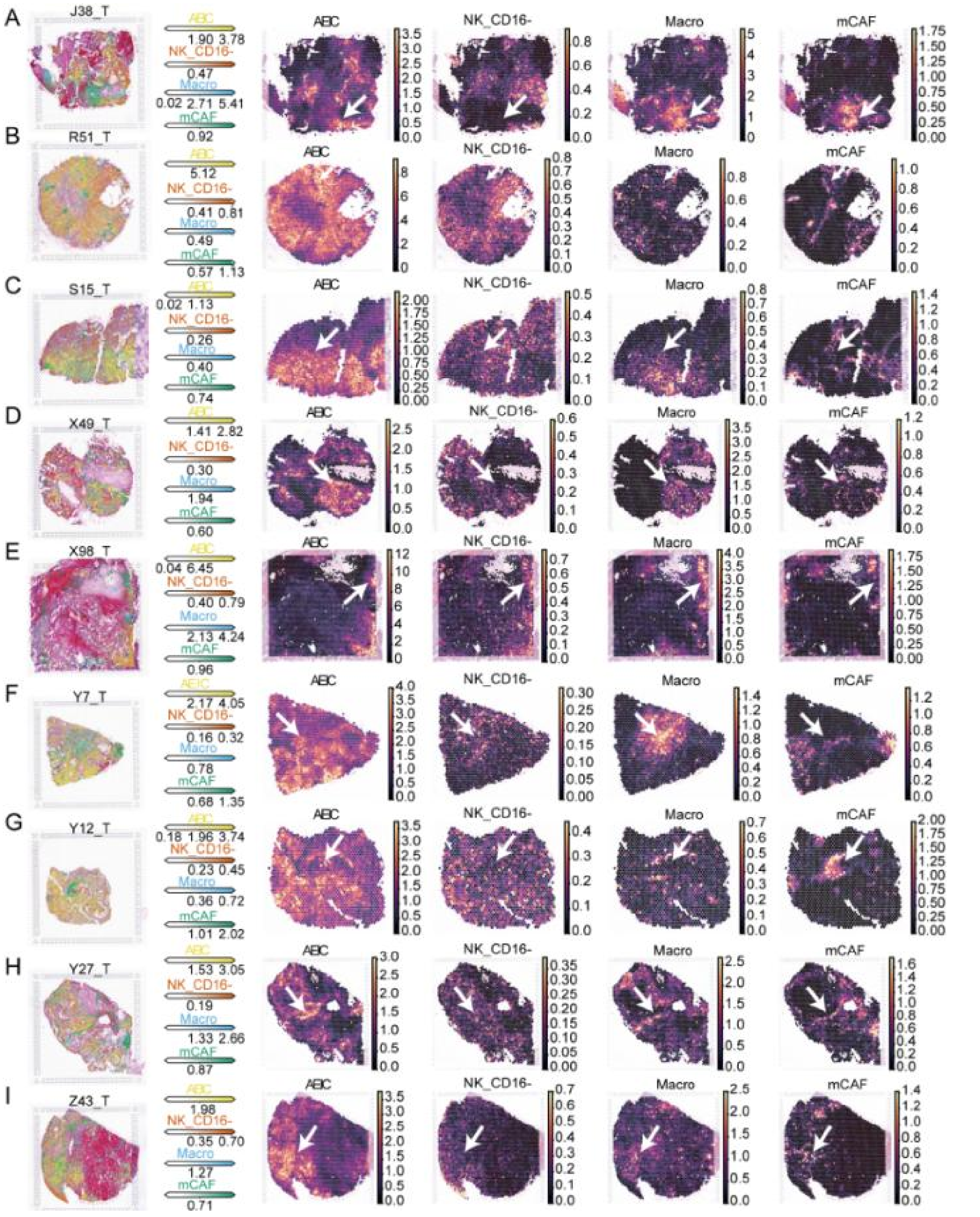

Figure S9. Spatial distribution of AEIC and microenvironmental cellular components across independent samples. (A-I) Spatial abundance mapping plots showing the in situ localization and infiltration patterns of AEIC, NK_CD16-, Macro, and mCAF cells across nine independent Visium spatial transcriptomic tissue sections, including J38_T (A), R51_T (B), S15_T (C), X49_T (D), X98_T (E), Y7_T (F), Y12_T (G), Y27_T (H), and Z43_T (I). Color bars represent estimated cell abundance values.

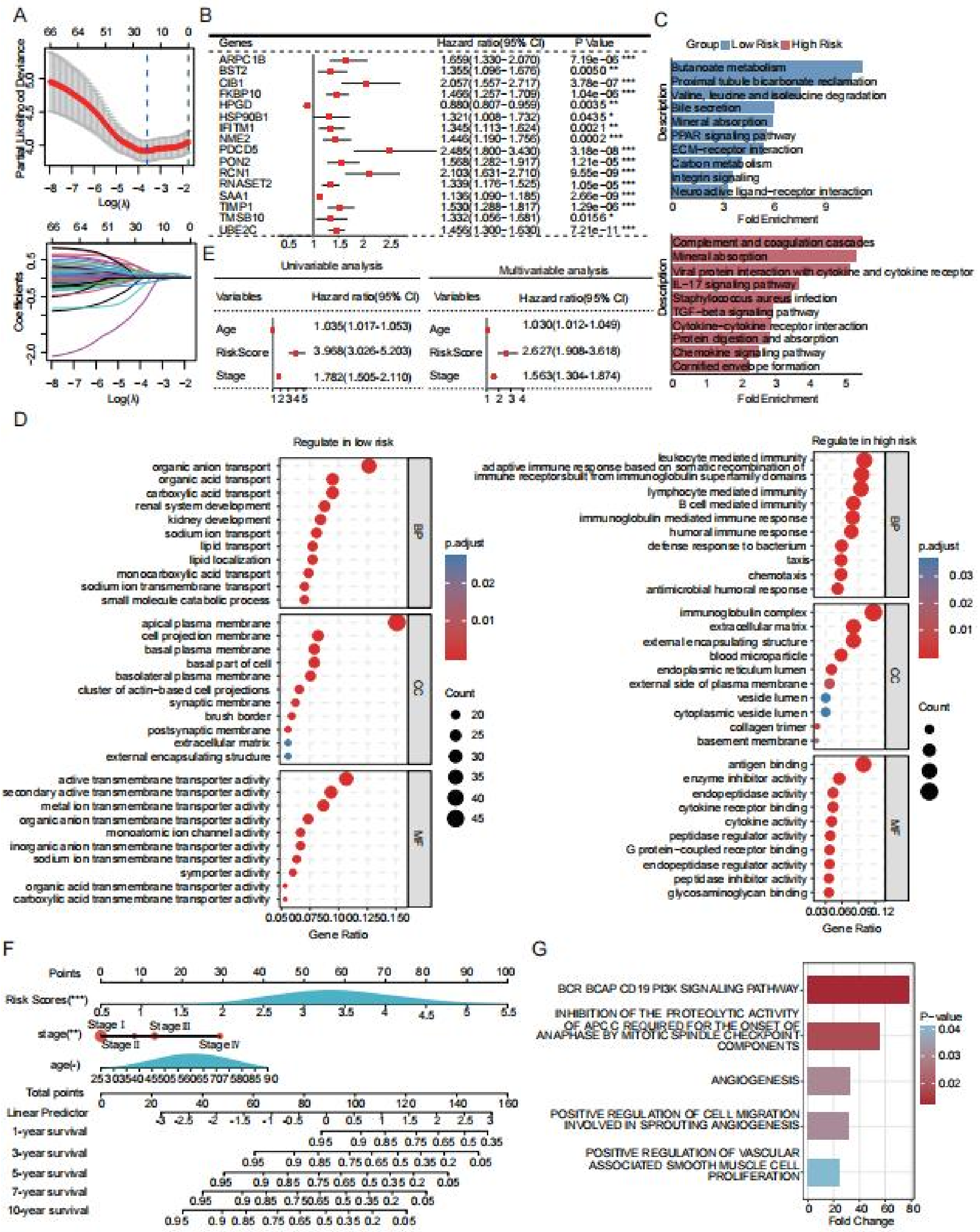

Figure S10. Construction and functional characterization of the prognostic risk model. (A) LASSO Cox regression analysis for selecting core prognostic genes. Upper panel: partial likelihood deviance plotted against log(λ) via 10-fold cross-validation, with dashed vertical lines indicating the optimal λ values. Lower panel: LASSO coefficient profiles of the 16 model-selected risk genes as a function of log(λ). **(B)** Forest plot showing univariate Cox regression analysis evaluating hazard ratios (HR) and 95% confidence intervals (CI) for the 16

AEIC-related risk genes (*P < 0.05, **P < 0.01, ***P < 0.001). **(C)**) Bar plots showing the top 10 significantly enriched KEGG pathways for downregulated genes in the low-risk group (top) and upregulated genes in the high-risk group (bottom). **(D)** Dot plots showing enriched GO terms across Biological Process (BP), Cellular Component (CC), and Molecular Function (MF) categories in the low-risk (left) and high-risk (right) groups. Circle size indicates gene count, and color intensity corresponds to the adjusted P value. **(E)** Forest plots of univariable (left) and multivariable (right) Cox regression analyses evaluating the independent prognostic value of clinical parameters (Age, Stage) and the risk score. **(F)** Nomogram integrating the risk score and clinical independent prognostic factors (Age, Stage) to predict 1-, 3-, 5-, 7-, and 10-year overall survival (OS). *(G)* Bar plot showing pathway enrichment of the 16 signature genes. Bar length represents fold change, and color gradient corresponds to P value.

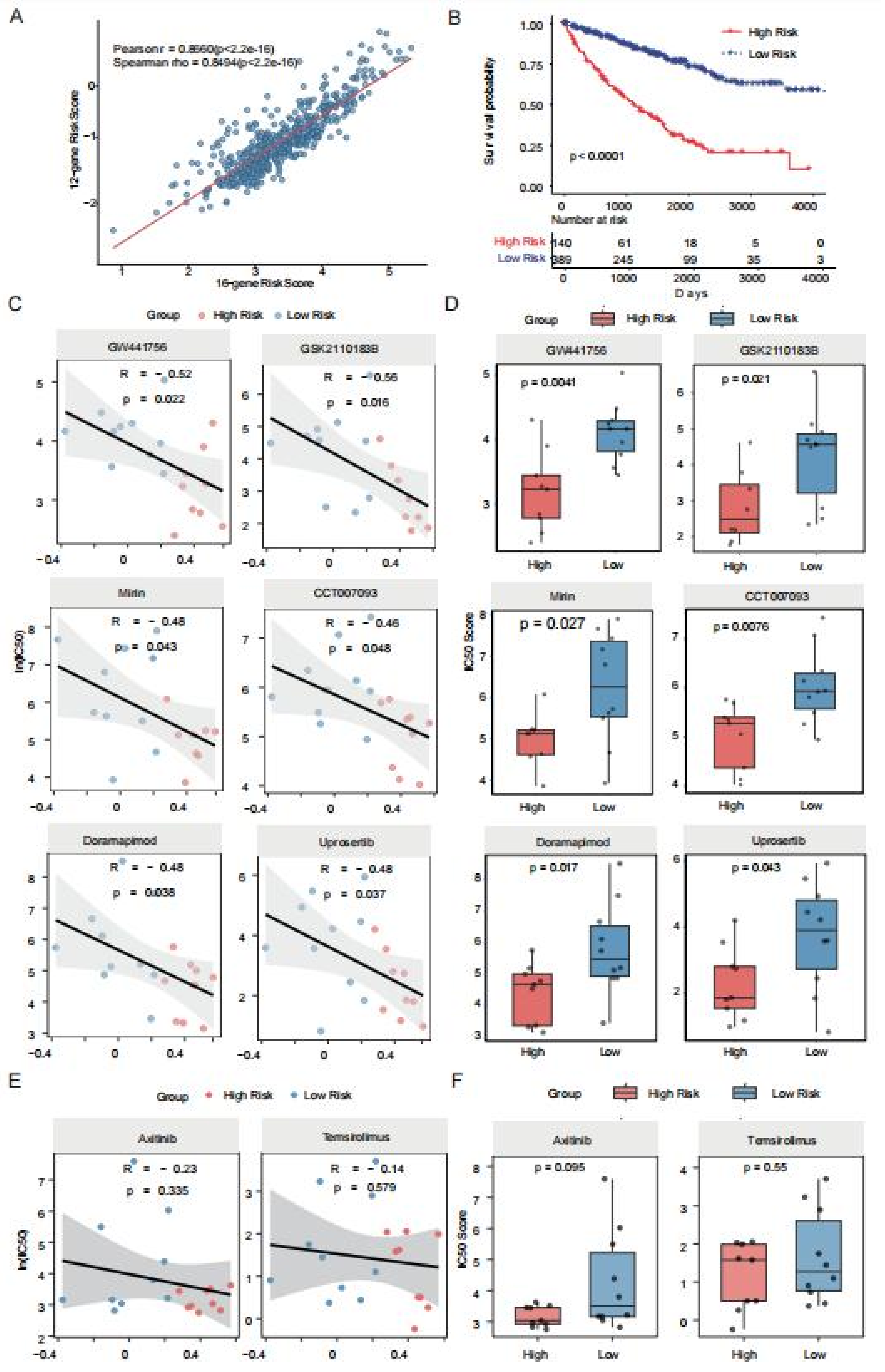

Figure S11. Robustness validation and exploratory drug-sensitivity analysis of the AEIC-derived prognostic signature. (A) Scatter plot showing the correlation between the original 16-gene RiskScore and the 12-gene RiskScore (reconstructed using GDSC2-available

signature genes) in the TCGA-KIRC cohort. Pearson and Spearman correlation coefficients are indicated. **(B)** Kaplan – Meier overall survival (OS) curves for TCGA-KIRC patients stratified into High Risk and Low Risk groups based on the 12-gene RiskScore ($P < 0.0001$, log-rank test). **(C)** Scatter plots showing Spearman rank correlation analysis between cell line risk scores and estimated ln(IC50) values for six selected therapeutic compounds in the GDSC2 dataset. **(D)** Boxplot comparisons of IC50 values for six drugs between the high-risk (red) and low-risk (blue) ccRC cell line groups. **(E)** Scatter plots showing Spearman rank correlations between cell line risk scores and estimated ln(IC50) values for established ccRCC therapies (Axitinib and Temsirolimus). **(F)** Box plots comparing IC50 values of Axitinib and Temsirolimus between High Risk (red) and Low Risk (blue) ccRCC cell line groups.
